# MgAl-Layered Double Hydroxide-Coated Bio-Silica as an Adsorbent for Anionic Pollutants Removal: A Case Study of the Implementation of Sustainable Technologies

**DOI:** 10.3390/ijms252111837

**Published:** 2024-11-04

**Authors:** Muna Abdualatif Abduarahman, Marija M. Vuksanović, Nataša Knežević, Katarina Banjanac, Milena Milošević, Zlate Veličković, Aleksandar Marinković

**Affiliations:** 1 Faculty of Technology and Metallurgy, University of Belgrade, Karnegijeva 4, 11120 Belgrade, Serbia; munsabdalla@gmail.com (M.A.A.); marinko@tmf.bg.ac.rs (A.M.); 2 Faculty of Science, University of Sabratha, Sabratha 240, Libya; 3 “VINČA” Institute of Nuclear Sciences—National Institute of the Republic of Serbia, University of Belgrade, Mike Petrovića Alasa 12-14, 11351 Belgrade, Serbia; natasa.knezevic@vin.bg.ac.rs; 4 Innovation Center of Faculty of Technology and Metallurgy Ltd., Karnegijeva 4, 11120 Belgrade, Serbia; kbanjanac@tmf.bg.ac.rs; 5 Institute of Chemistry, Technology and Metallurgy—National Institute of the Republic of Serbia, University of Belgrade, Njegoševa 12, 11000 Belgrade, Serbia; milena.milosevic@ihtm.bg.ac.rs; 6 Military Academy, University of Defense, Veljka Lukića Kurjaka 33, 11000 Belgrade, Serbia; zlatevel@yahoo.com

**Keywords:** MgAl-LDH, adsorption, enzymatic decolorization, silica reinforcement, UPR composites

## Abstract

The adsorption efficiency of Cr(VI) and anionic textile dyes onto MgAl-layered double hydroxides (LDHs) and MgAl-LDH coated on bio-silica (b-SiO_2_) nanoparticles (MgAl-LDH@SiO_2_) derived from waste rice husks was studied in this work. The material was characterized using field-emission scanning electron microscopy (FE-SEM/EDS), X-ray diffraction (XRD), Fourier transform infrared spectroscopy (FTIR), and X-ray photoelectron spectroscopic (XPS) techniques. The adsorption capacities of MgAl-LDH@SiO_2_ were increased by 12.2%, 11.7%, 10.6%, and 10.0% in the processes of Cr(VI), Acid Blue 225 (AB-225), Acid Violet 109 (AV-109), and Acid Green 40 (AG-40) dye removal versus MgAl-LDH. The obtained results indicated the contribution of b-SiO_2_ to the development of active surface functionalities of MgAl-LDH. A kinetic study indicated lower intraparticle diffusional transport resistance. Physisorption is the dominant mechanism for dye removal, while surface complexation dominates in the processes of Cr(VI) removal. The disposal of effluent water after five adsorption/desorption cycles was attained using enzymatic decolorization, photocatalytic degradation of the dyes, and chromate reduction, satisfying the prescribed national legislation. Under optimal conditions and using immobilized horseradish peroxidase (HRP), efficient decolorization of effluent solutions containing AB-225 and AV-109 dyes was achieved. Exhausted MgAl-LDH@SiO_2_ was processed by dissolution/precipitation of Mg and Al hydroxides, while residual silica was used as a reinforcing filler in polyester composites. The fire-proofing properties of composites with Mg and Al hydroxides were also improved, which provides a closed loop with zero waste generation. The development of wastewater treatment technologies and the production of potentially marketable composites led to the successful achievement of both low environmental impacts and circular economy implementation.

## 1. Introduction

Water pollution, resulting from industrial activities, necessitates the continuous development of new, tailored wastewater treatment methods. Pollutants, such as heavy metals and organic compounds, e.g., pharmaceuticals1, pesticides, dyes, etc., are undesirable pollutants. The widely recognized toxic and carcinogenic effects of Cr(VI) [1] underscore its significant impact on human health [2]. Chromium, frequently present in industrial discharge from sectors like chemicals, paints, metal finishes, stainless steel manufacturing, alloy cast irons, chrome, and wood treatment, poses a significant threat due to its high mobility in ecosystems and adverse effects on human health [3]. Hexavalent chromium in aqueous systems exists in various pH-dependent oxoanionic forms, including hydrogen chromate (HCrO^4−^), chromate (CrO_4_^2−^), and dichromate (Cr_2_O_7_^2−^) [4], that are linked to severe health conditions, such as lung cancer, nasal irritation, nasal ulcers, hypersensitivity reactions, and contact dermatitis [5]. Recognizing the health risks, the World Health Organization (WHO) has set a maximum allowable concentration of Cr(VI) in drinking water at 0.05 mg L^−1^.

Organic dyes play a pivotal role in industries such as paper, paint, plastic, and textiles, featuring intricate molecular structures like azo, anthraquinonoid, and heterocyclic groups; however, due to their extensive application and resistance to degradation, they pose significant hazardous potential [6]. Their environmental persistence results in their accumulation in the environment, leading to contamination of food chains as potential threats to humans [7], and the combined presence of heavy metal ions and dyes generally imparts greater toxicity with respect to living organisms [8].

The current challenge in wastewater treatment lies in the coexistence of different pollutants, including anionic [9] and cationic [10] pollutants, markedly amplifying the difficulty and cost of water treatment. Conventional technologies face inefficiencies in simultaneously removing these diverse pollutants due to their distinct physicochemical properties, including their molecular size and chemical structure [11]. Therefore, it becomes imperative to devise effective approaches for the removal of coexisting pollutants.

Various techniques have been explored, including electrochemical precipitation [12], ion exchange [13], membrane ultrafiltration [14], and adsorption. Adsorption stands out as a cost-effective technique. Additionally, when coupled with an effective desorption process, adsorption can address the sludge-related challenges commonly encountered in precipitation methods. Various adsorbents, including clay [15], zeolite [16], carbon-based materials [17], and layered double hydroxide (LDH), have been used [18].

Layered double hydroxides represent a versatile class of two-dimensional (2D) inorganic layered matrices, attracting considerable attention owing to their distinctive physical and chemical properties. These properties have been translated into outstanding performance across diverse applications, including catalysis [19], photochemistry [20], electrochemistry [21], biotechnology [22], medicine [23], adsorption in wastewater treatment [24], and support for enzyme immobilization due to good enzyme retention capacity [25]. LDH helps in the preservation of enzyme activity and supports charge transport in the immobilized system [26]. Three approaches, including coprecipitation methods, direct exchange methods, and rehydration methods, are commonly applied for LDH synthesis [27].

The main idea and novelties of the study are reflected in the development of sustainable water purification technologies that result in the minimization of negative environmental impacts. Using the 3R approach (reduce, reuse, recycle), bio-based materials replaced commercial ones, adsorbents were reused, and spent adsorbents were repurposed. The use of silica (SiO_2_) from waste rice husk as a support for MgAl layered double hydroxides precipitation (LDHs are well-known adsorbents for anionic pollutants removal from water) was used to improve the applicability and adsorption performance of newly synthesized MgAl-LDH@SiO_2_ adsorbent. Efficient water purification, desorption, and the proper disposal of effluent desorption water and discharged adsorbent into valuable materials were achieved. Adsorption studies were performed in relation to isotherm, kinetic, and thermodynamic performances in a batch system at moderate and low initial pollutants concentration. An adsorption/desorption study in a flow system with subsequent environmentally friendly technologies developed for the treatment of effluent waters was proposed. Cr(VI) was transformed to solidified material, AG-40 was subjected to photocatalytic decomposition, and the decolorization of effluent water containing AB-225 and AV-109 dyes using immobilized horseradish peroxidase (HRP) was performed. All of the parameters of the treated water were below the values prescribed by the national regulations, as outlined in the “Official Gazette of RS” nos. 67/2011 and 48/2012, regarding limit values of the emission of pollutants and deadlines for achieving them, as well as the Water Framework Directive of the European Commission [28]. The implementation of the principles of sustainable development was realized in a novel way by either providing valuable products or minimizing the negative environmental impacts of discharged treated water.

## 2. Results and Discussion

### 2.1. Characterization of MgAl-LDH and MgAl-LDH@SiO_2_ Particles

#### 2.1.1. Morphological Study

**Figure 1:**
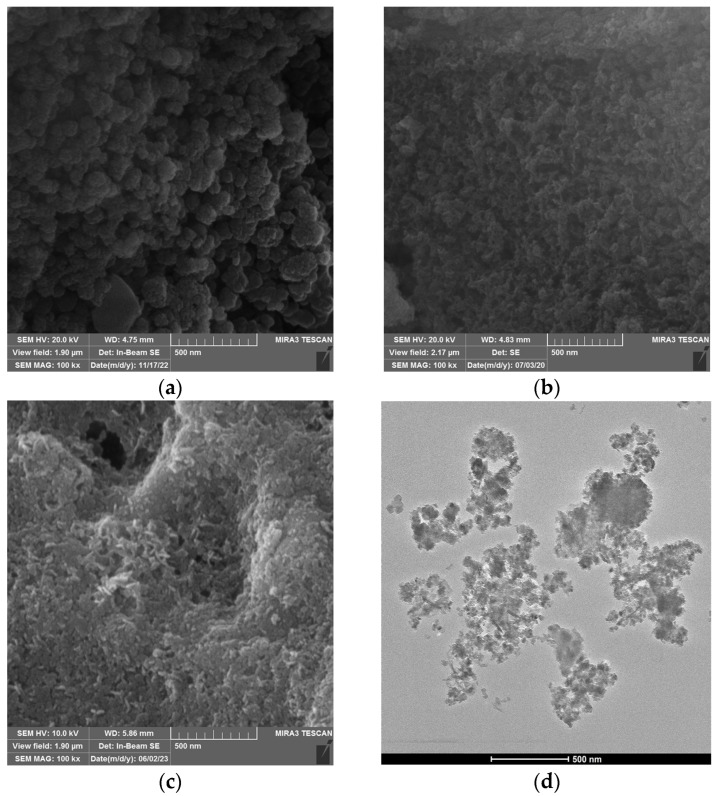
Morphology of (**a**) SiO_2_, (**b**) MgAl-LDH, (**c**) and MgAl-LDH@SiO_2_ particles and (**d**) TEM images of MgAl-LDH@SiO_2_.

The morphological features of bio-silica, MgAl-LDH, and MgAl-LDH@SiO_2_ particles were analyzed according to results of SEM microscopy (Figure 1).

Figure 1a shows that the silica particles have an irregular shape, while the MgAl-LDH and MgAl-LDH@SiO_2_ particles are in the form of flakes (Figure 1b,c).

**Figure 2:**
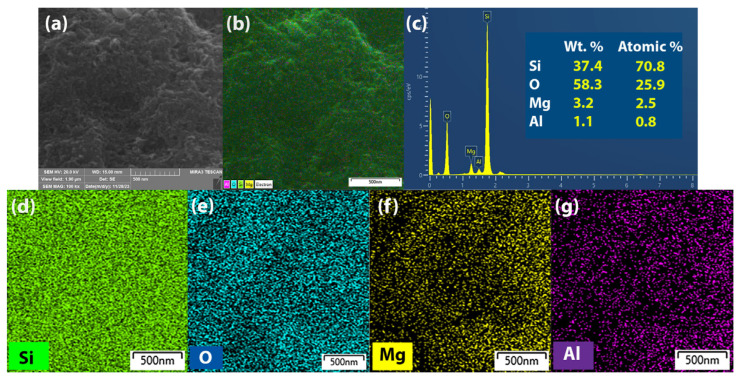
(**a**) SEM images of MgAl-LDH@SiO_2_ particles, (**b**) merged image of MgAl-LDH@SiO_2_, (**c**) EDS mapping results, and (**d**–**g**) elemental mapping of Si, O, Mg, and Al.

A mapping image of the elemental composition of MgAl-LDH@SiO_2_ particles was obtained from EDS analysis on a significant portion of the SEM sample (Figure 2 and Figure S1). The EDS analysis (Figure 2) shows that the elemental content is as follows: Si 70.8%, O 25.9%, Mg 2.5%, and Al 0.8%. The diameter distribution of MgAl-LDH@SiO_2_ (Figure S2) indicates that the mean diameter is 42 ± 9 nm.

#### 2.1.2. XRD and FTIR Structural Characterization

**Figure 3:**
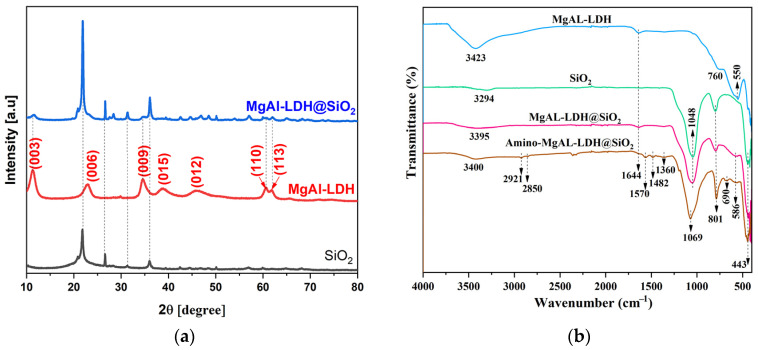
(**a**) XRD patterns of bio-silica, MgAl-LDH, and MgAl-LDH@SiO_2_ particles and related (**b**) FTIR spectra.

The XRD pattern and FTIR spectra of bio-silica, MgAl-LDH, and MgAl-LDH@SiO_2_ are given in Figure 3. In addition, FTIR spectra of MgAl-LDH@SiO_2_ after dyes adsorption are given in Figure S3.

The SiO_2_ diffraction pattern displays the amorphous silica’s reflecting property [29]. The MgAl-LDH diffraction peaks correspond to planes (003), (006), (009), (015), (012), (110), and (113), which indicate a layered structure. Because the MgAl-LDH@SiO_2_ was synthesized intentionally with 7.6 wt.% of LDH deposit on low-crystalline SiO_2_, the peaks for the LDH structure are smaller in Figure 3a. In addition, because the layer is small, the crystals are also small [30].

Two bands, observed at 3423 and 1644 cm^−1^ in the FTIR spectrum of MgAl-LDH (Figure 3b), are related to the stretching and bending vibrations of hydroxyl groups in the MgAl hydroxide layer and water in the interlayer, respectively. Vibrations of the Mg-O and Al-O groups were noticed in the range 550–760 cm^−1^ [31]. After coprecipitation at b-SiO_2_, the new peaks observed at 1048, 801, 586, and 443 cm^−1^ are assigned to the stretching vibrations of Si-O-Si, Si-O, Mg-O-Mg/Al-O-Al/Si-O-Si, and O-Si-O in MgAl-LDH@SiO_2_, respectively [31,32]. The spectrum of amino-MgAl-LDH@SiO_2_ showed additional bands at 2921–2850 and 1482–1360 cm^−1^ related to C-H stretching and the deformation of methyl and methylene groups. In addition, bands at 1570 and 690 cm^−1^ were assigned to N-H bending and deformations of amino groups, respectively.

#### 2.1.3. XPS Analysis

**Figure 4:**
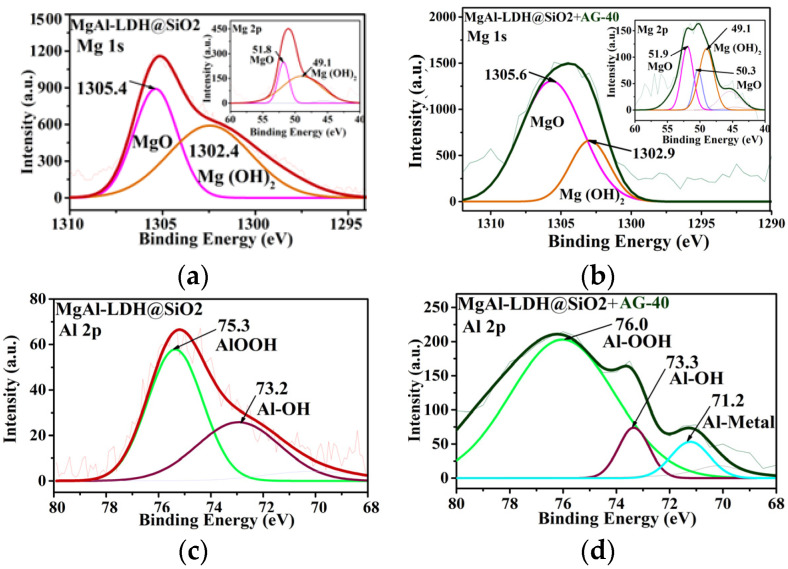

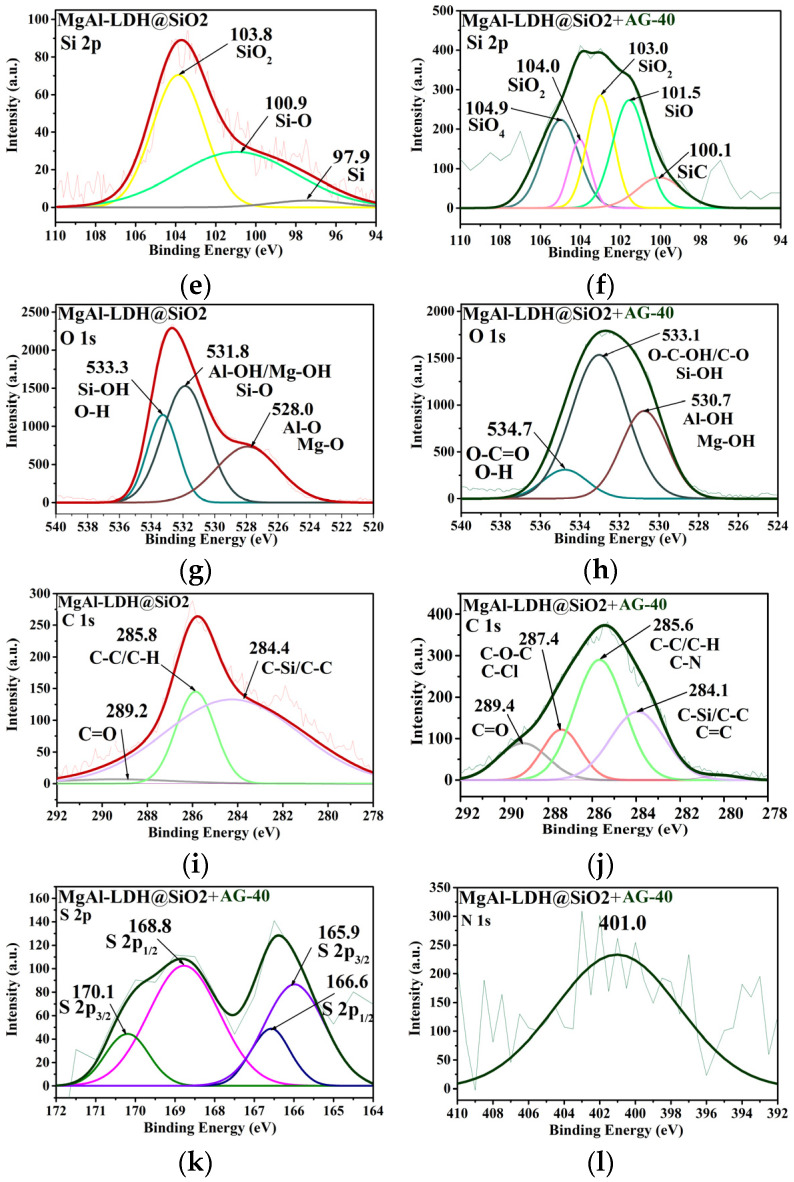
The core level XPS spectra of (**a** and **b**) Mg 1s, Mg 2p, (**c** and **d**) Al 2p, (**e** and **f**) Si 2p, (**g** and **h**) O 1s, (**i** and **j**) C 1s, (**k**) S 2p, and (**l**) N 1s of the MgAl-LDH@SiO_2_ and MgAl-LDH@SiO_2_ + AG-40.

The XPS spectra of the MgAl-LDH@SiO_2_ adsorbent before and after adsorption are given in Figure 4 and Figures S4–S6, respectively. The survey spectrum of MgAl-LDH@SiO_2_ (Figure S4) displayed the presence of Mg, O, C, Al, and Si in 1s, 2s, and 2p orbit states, and two additional S 2p and N 1s XPS signals are observed in the survey spectra after dye adsorption. The Mg 1s, Mg 2p, and Al 2p spectra (Figure 4a,c) demonstrate the presence of Mg^2+^ (1302.4 and 1305.4 eV), Mg^2+^ (49.1 and 51.8 eV), and Al^3+^ (73.2 and 75.3 eV) valent states, respectively, corresponding to Mg(Al)-OH/Mg(Al)-O [33]. In addition, the Si 2p spectrum (Figure 4e) is characterized by three deconvoluted peaks at 97.9, 100.9, and 103.8 eV binding energy, which are related to elementary Si and its oxide [33]. The O 1s spectrum (Figure 4g) was deconvoluted into three overlapping peaks at 528.0, 531.8, and 533.3 eV, associated with Mg(Al)-O, Mg(Al)-OH/Si-O, and Si-OH/OH(adsorbed H_2_O) eV, [31] respectively, while the small C1s spectrum (Figure 4i) was deconvoluted into three peaks at 284.4, 285.8, and 289.2 eV corresponding to C-Si/C-C, C-C/C-H, and C=O functional groups, respectively.

In the spectra after adsorption (Figure 4, Figures S5 and S6), a shift in the binding energy and a change in the shape and intensity of the deconvolution peaks were observed. In addition, the appearance of new peaks in the following spectra were noticed: Mg 2p at 50.3 eV (Figure 4b) and 50.9 (Figures S5a and S6c); Al 2p at 71.2 eV (Figure 4d), 72.7/77.7 eV (Figure S5b), and 72.9eV (Figure S6b); and Si 2p at 100.1/104.0/104.9 eV (Figure 4f), 104.8 eV, (Figure S5c) and 102.4 eV (Figure S6c) attributable to Mg(Al, Si)-O, Al-Metal, and Al-O. This indicates that these functional groups that contain Mg/Al/Si participate in the interactions between the adsorbent and dye.

Further, in the O 1s spectra after adsorption, a notable decrease in the intensity of the deconvoluted peaks is observed, and the peaks are significantly shifted to higher binding energy values, suggesting an interaction between dyes and sorbent. The peaks at 530.7, 533.1, and 534.7 eV after AG-40 adsorption (Figure 4h); 531.0, 533.3, and 534.9 after AB-225 adsorption (Figure S5d); and 531.0, 532.9, and 534.2 after AV-109 adsorption (Figure S6d) are attributed to Mg(Al)-hydroxide, Si-hydroxide/O-C-OH/C-O, and O-C=O/OH (adsorbed H_2_O), respectively [31,33]. Deconvolution of the C1s spectrum of MgAl-LDH@SiO_2_ after adsorption indicates the presence of considerable functional groups from the dyes, including C-N, (285.6 eV) (Figure 4j, Figures S5e and S6e), N-C=O (288.2 eV) (Figures S5e and S6e), C-Cl (287.4 eV) (Figure 4j), C-Br (286.2/286.3 eV) (Figures S5e and S6e), and C=C from the aromatic structure of dyes at ~284 eV, which overlaps with C-Si from the sorbent (Figure 4j, Figures S5e and S6e). Finally, the S2p [31,32] in the spectra of all adsorbent samples can be fitted by four deconvoluted peaks (Figure 4k, Figures S5g and S6g), and a peak for N 1s around ~400 is also observed (Figure 4l, Figures S5f and S6f). These results indicate the interaction of the sulfonate groups from the dyes with the MgAl-LDH@SiO_2_ surface.

#### 2.1.4. Determination of Zero Point Charge (pH_PZC_)

The values of pH_pzc_ at different ionic strengths (Figure S7) were found to be 7.9 and 8.1 for MgAl-LDH and MgAl-LDH@SiO_2_, respectively. The extent of positive charge at pH < pH_pzc_ at each adsorbent surface depends on operative pH and surface properties, but has a low dependence on ionic strength. This parameter strongly indicates the high applicability of both adsorbents for anionic pollutant removal at pH < pH_pzc_.

### 2.2. Adsorption Studies

#### 2.2.1. Adsorption Isotherm Study

The results of the processing of adsorption data using the Langmuir (Equation (S1)) and Freundlich isotherm models (Equation (S2)) are given in Table 1, Tables S1–S3 and Figure S8 [9].

The capacities of single-layer coverage (q_m_) for chromate and dyes increase as the temperature increased (Table 1) and confirm the high applicability of MgAl-LDH@SiO_2_ for anionic pollutant removal. The results obtained indicate that the synergetic effect of the specific morphology of b-SiO_2_, despite its lower surface area, combined with the deposition of MgAl-LDH, provides an active surface. This configuration offers a high availability of surface-active sites able to interact with anionic pollutants (see Section S3.2.2). The comparative study of the adsorption results for MgAl-LDH@SiO_2_ (Table 1) and MgAl-LDH (Table S1) showed an almost linear relationship in q_m_ values for these two adsorbents, demonstrating the value of the applied methodology. In addition, the results demonstrate an appropriate relation between q_m_ and the structural properties of the dyes, specifically the availability of the basic/proton donating sites. This indicates a higher availability of the sulfonate group in AG-40 dye for interactions with adsorbent’s surface charge and functionalities (Figure S8). Otherwise, higher steric hindrance of the neighboring groups in AB-225 and AV-109 contribute to the decrease in the sulfonate group availability. Additionally, the adsorption study results at C_i_ = 1 mg dm^−3^ are given in Table S4.

#### 2.2.2. Thermodynamic Study

To analyze the thermodynamic aspect of the adsorption process, the Gibbs free energy (ΔG^Θ^), enthalpy (ΔH^Θ^), and entropy (ΔS^Θ^) were calculated using Van’t Hoff equations, i.e., Equations (S3) and (S4) [34]. The obtained results are given in Table 2 and Table S5.

The negative ΔG^Θ^ values at all temperatures demonstrate the feasibility and spontaneity of the adsorption processes (Table 2 and Table S5). The dependence of ΔG^Θ^ and ΔH^Θ^ values versus temperature confirms more effective desolvation of the studied ion/dyes and diffusional processes. Low influences of the structural and physico-chemical properties of Cr(VI) oxyanions and dye molecules on the state of equilibrium can be observed.

#### 2.2.3. Adsorption Kinetics

Process kinetics were analyzed using pseudo-first order (PFO), pseudo-second order (PSO), i.e., the Ho–Mackay model, and a second-order model [35], and the results of the statistically reliable PSO model fitting and the activation energy (Ea) are shown in Table 3 and Table S6.

Diffusional resistance was evaluated by fitting kinetic data with the Weber–Morris (W–M) model, the Dunwald–Wagner model (D-W), and the homogeneous diffusion model (HSDM) [9] (Table 4 and Table S7). The LDH structure exhibits a gallery pathway that facilitates carrier diffusion and transportation throughout the entire particle bulk [36].

#### 2.2.4. Continuous Flow Experiments

The calculated column parameters (see theoretical background in S2.2.4) using the Bohart–Adams (B–A) [37] and Yoon–Nelson (Y–N) [38] models are given in Table 5, Tables S8 and S9 and Figure S9.

### 2.3. Desorption Study in a Column System

Adsorption/desorption cyclability provides a relevant indicator of adsorbent longevity and cost-effectiveness, which are crucial criteria for assessing potential applicability. The type and strength of adsorbate/adsorbent interactions, as well as the regenerant’s displacement power, primarily govern the efficacy of adsorption/desorption processes. Meanwhile, the choice of regenerant dictates the degree of material erosion.

Due to sensitivity of the MgAl-LDH deposit to strong acidic medium, a brief study related to regenerant type and desorption condition selection (desorption efficiency versus concentration and time) was performed (SM S2.3). Accordingly, two adsorption/desorption technologies were developed:

- A method using a moderate dye inlet concentration (25 mg dm^−3^) (S2.4.2) with a low volume of regenerator (Table S10) was developed to promote dye regeneration (less favorable).
- A method using a low dye inlet concentration (1 mg dm^−3^) (Table 6) with a larger volume of desorption solution at higher desorption efficiency (Table S10) was developed with potential for wastewater purification (highly favorable).

The establishment of circular technologies, after pollutant desorption, was achieved using different treatments of the effluent water: enzymatic decolorization of AV-109 and AB-225 (Section 2.4) as well as photocatalytic degradation of AG-40 (Section 2.5).

### 2.4. Decolorization of Wastewater Using Immobilized HRP on an Amino-MgAl-LDH@SiO_2_ Support

#### 2.4.1. Immobilization of HRP on an Amino-MGAl-LDH@SiO_2_ Support

**Figure 5:**
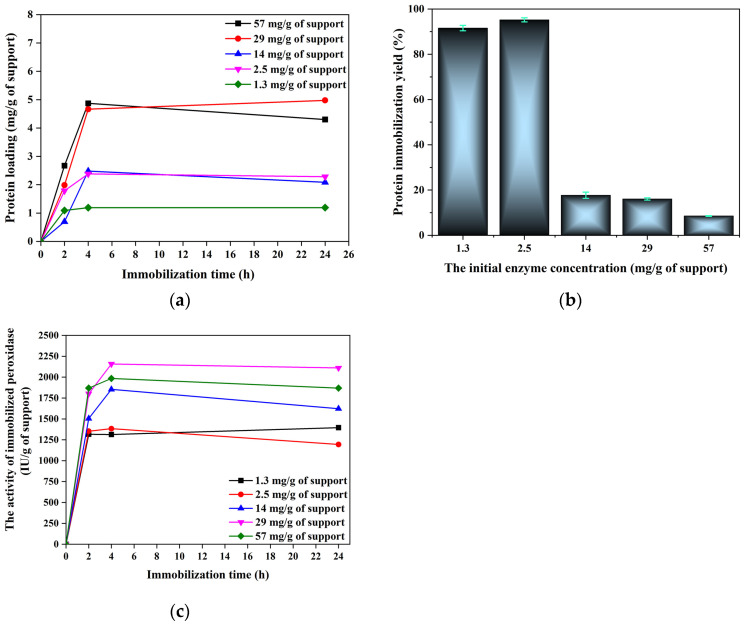
(**a**) Influence of the initial enzyme concentration on protein loading (mg/g of support) and (**b**) protein immobilization yield (%), and (**c**) the effect of immobilization time on the activity of immobilized peroxidase at different protein concentrations. The activity of immobilized HPR on amino-MgAl-LDH@SiO_2_ was determined based on a reaction with commercial substrate pyrogallol.

First, HPR immobilization on amino-modified MgAl-LDH@SiO_2_ particles was examined in order to determine if this support has potential for use for enzyme immobilization. Herein, the initial enzyme concentration was varied to examine the support capacity for enzyme attachment and the activity of immobilized preparations. The impact of the initial enzyme concentration on the mass of bound protein and the activity of immobilized peroxidase during the time period are presented in Figure 5.

Immobilization was performed at different initial enzyme concentrations, ranging from 1.3 to 57 mg/g of support. The enzyme was immobilized in a Na-phosphate buffer at pH 7 because, at this pH, the amino groups on the enzyme are expected to be positively charged. In contrast, the enzyme molecules will carry an overall negative charge at pH values above the isoelectric point. This difference in charge promotes attractive interactions between the enzyme molecules and the support [39].

Increasing the initial enzyme concentration up to 57 mg/g of support resulted in an increase in protein loading to 5 mg/g of support (Figure 5a). Immobilized peroxidase exhibited the lowest activity of 1189.6 IU/g after 24 h at an initial protein content of 1.3 mg/g of support (Figure 5b). The enzyme activity of the immobilized preparation was in the range of 1750 to 2158 IU/g at the initial protein concentrations above 14 mg/g of support, indicating that the optimal immobilization time is 4 h and the optimal initial enzyme concentration is 29 mg/g of support. The plot (Figure 5c) shows that at the optimal initial enzyme concentration of 29 mg/g of support, the immobilized enzyme expressed a maximum immobilized enzyme activity of 2158 IU/g of support, a protein immobilization yield of 15% (Figure 5b), and a specific activity of 431.6 IU/mg of protein (Table S11). The presented results have shown that HRP immobilized on mesoporous silica via adsorption exhibits higher activities at lower enzyme loadings, suggesting that amino-MgAl-LDH@SiO_2_ has the potential to be used as support for HPR immobilization.

#### 2.4.2. Activation of Support with Glutaraldehyde

After demonstrating that amino-MgAl-LDH@SiO_2_ can be used for the immobilization of HRP via adsorption, the amino group was activated with glutaraldehyde (GA) to obtain a more stable immobilized preparation by forming covalent bonds between the GA-activated support and the amino groups of enzyme molecules. For support activation, a 1% solution of GA was used (SM S3.5). The effect of the GA activation on protein loading and enzyme activity was examined (Table S11). The results indicated that activating the support led to a 22% decrease in activity and a 28% reduction in protein loading. However, activation of the support with GA did not result in the deactivation of the enzyme molecules during immobilization, which could have occurred [40], as evidenced by the fact that the specific activity remained unchanged after activation.

On the other hand, a desorption assay with 1 M CaCl_2_ and 1% triton demonstrated that covalent bonds between the enzyme molecules and GA activated amino-MgAl-LDH@SiO_2_ are formed. In the case of the HPR-immobilized preparation, 86% of all bonds formed between HPR and GA-amino-MgAl-LDH@SiO_2_ were be covalent. It can be presumed that the stable covalent immobilized preparation will have much more prospects for use in the decolorization of wastewater in comparison with the enzyme immobilized by adsorption.

#### 2.4.3. Decolorization Efficiency and Reusability Study

**Figure 6:**
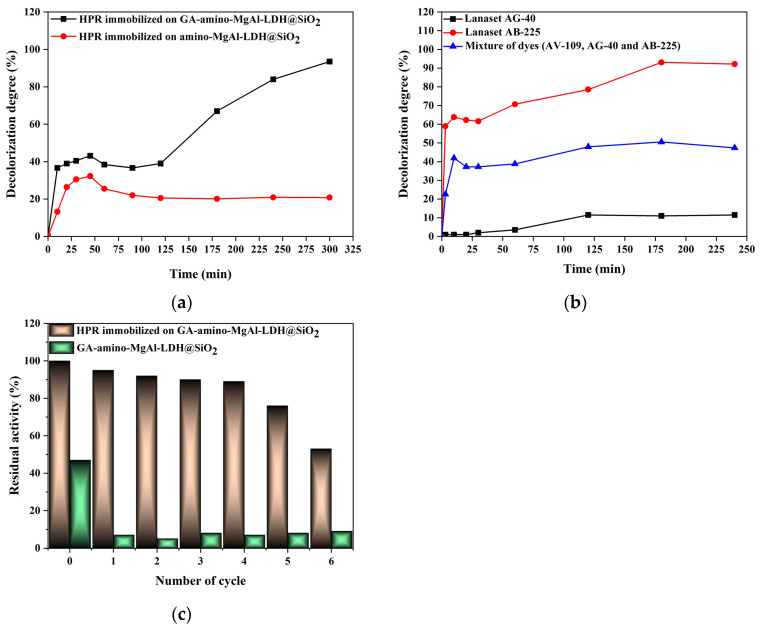
(**a**) Decolorization efficiency of HPR immobilized on amino-MgAl-LDH@SiO_2_ and GA-amino-MgAl-LDH@SiO_2_. (**b**) Decolorization of AG-40 and AB-225 dye and a mixture of dyes using HPR-immobilized GA-amino-MgAl-LDH@SiO_2_ support. (**c**) Reusability of the produced GA-amino-MgAl-LDH@SiO_2_-HPR preparation in the decolorization of the textile dye AV-109 and the adsorption of AV-109 dye on GA-amino-MgAl-LDH@SiO_2_.

To fully exploit the potential of the immobilized peroxidase produced in the decolorization reaction, the decolorization of AV-109 dye was conducted using both GA-activated and non-activated supports (Figure 6a). The decolorization efficiency of the obtained HPR-immobilized preparations was examined (Figure 6a) under reaction conditions of the textile dye AV-109 at a concentration of 25 mg L^−1^ and pH 4.0 in the presence of H_2_O_2_ (concentration of 0.08%) [41] (Table S12). The anthraquinone dye AV-109 was chosen as the model dye in order to preliminary determine if the immobilized preparations could be used for wastewater treatment. Subsequently, AG-40 dye, AB-225 dye, and a mixture of dyes (AV-109, AG-40, AB-225) were treated with HPR covalently immobilized on GA-amino-MgAl-LDH@SiO_2_ (Figure 6b).

The highest decolorization efficiency (90%) was achieved within 300 min with HPR immobilized on GA-activated support (GA-amino-MgAl-LDH@SiO_2_) (Figure 6a). On the other hand, degradation with HPR immobilized on amino-MgAl-LDH@SiO_2_ remained stable after 50 min of treatment, with a maximum decolorization efficiency of only 30%.

Preliminary experiments showed that the adsorption capacity of both supports is up to a maximum of 50% for 5 h in the case of the AV-109 dye. This indicates that the greater than 50% decolorization efficiency of the immobilized enzyme could be ascribed only to the enzyme’s activity, with the highest impact observed after 125 min in case of the HPR immobilized on the GA-activated support.

A comparison of the results for GA-activated and amino supports clearly demonstrates that activation with GA positively impacted the affinity of peroxidase toward AV-109 decolorization. Therefore, the decolorization potential of HPR immobilized on GA-activated support was examined in reactions with two more anthraquinone dyes as well as a mixture of dyes under similar reaction conditions as noted for the AV-109 dye.

The results showed that GA-amino-MgAl-LDH@SiO_2_ is capable of decolorizing the AB-225 dye by 92% within 175 min (Figure 6b), while it decolorized the mixed dye solution by 49% in the same timeframe. For AG-40 dye, a low decolorization efficiency of ~10% over 240 min was detected. Based on the presented results, it can be concluded that HPR immobilized on GA-amino-MgAl-LDH@SiO_2_ could be used for the degradation of AV-109 and AB-225 with high efficiency. In the case of the dye mixture, the efficiency is approximately 50% due to the presence of AG-40. The poor performance of the HPR immobilized preparation with AG-40 dye can be attributed to the dye’s chemical structure, which affects its ability to approach the enzyme’s active site effectively. Since HPR is unable to degrade AG-40 dye, photodegradation will be applied.

Based on reusability research, the potential for using the derived biocatalyst on an industrial scale was also assessed. The findings are displayed in Figure 6c.

The biocatalyst applied under optimal reaction conditions was separated from the reaction mixture after each cycle, and its remaining catalytic activity was assessed in comparison to the first cycle (100%). Over the course of five cycles, the HRP immobilized preparation maintained a decolorization efficiency above 90%. However, a gradual decrease in decolorization efficiency was noted in the 5th and 6th cycles. In contrast, for the GA-activated support, dye adsorption was observed only after the initial cycle. Given that 50% of the dye is adsorbed after the initial cycle and only 5% is adsorbed in the subsequent five cycles, it can be concluded that the performance of immobilized HPR preparation in dye decolorization during reuse is solely due to the enzyme.

### 2.5. Photodegradation of Effluent Water Containing AG-40

**Figure 7:**
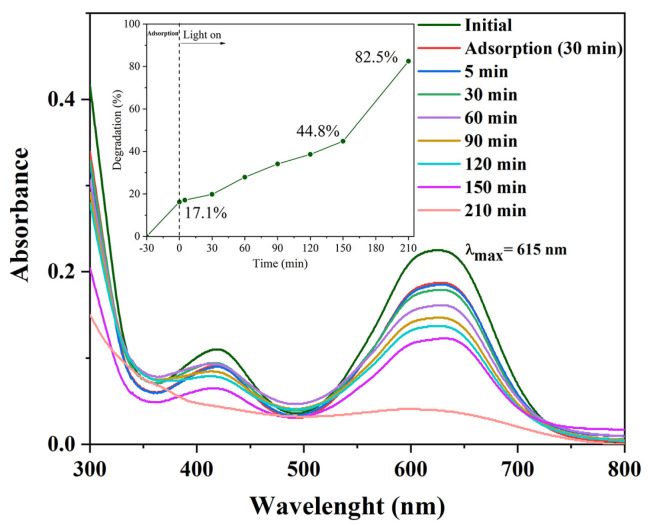
Time-dependent UV spectra in the course of the photodegradation of AG-40 dye.

Photocatalytic tests were conducted using zinc oxide at 0.08 g L^−1^ and an initial dye concentration (effluent solution, Table 6) of 22.7 mg/L for 210 min (S2.5). The decrease in absorbance at 615 nm versus time is given in Figure 7.

The efficiency of photodegradation, demonstrating a rate of 82.5% after 210 min, depends on the reaction conditions, dye structure, and irradiation efficiency [42]. The rate of the photocatalytic reaction is determined by pseudo-first-order kinetics: k_1_ = 0.0073 min^−1^ and t_1/2_ = 94.9 min [43,44]. The determination of the COD value was performed to evaluate the potential environmental threat of the effluent and treated water (S2.5) providing one general parameter of water quality (Table S13). The trend of COD values showed a nearly linear decrease, reaching 178 mg O_2_/L after 210 min and 126 mg O_2_/L after 4 h of irradiation. Both values are lower than 200 mg O_2_/L, as prescribed by the Serbian national regulation on sewage water from the textile industry (S2.5). Results from photolysis experiments and quantum yield determination (S2.5; Table S14) confirm the photocatalytic degradation potential for future optimization in real water. The effective treatment and purification of water provide effluent waters that are able to be safely discharged into water-courses.

### 2.6. Recycling of Exhausted MgAl-LDH@SiO_2_

Exhausted MgAl-LDH@SiO_2_ particles were transformed into a native form of bio-silica through acid washing and used as reinforcements in b-UPR [45]. A SEM micrograph of recycled bio-silica is given in Figure S10. Acidic washing was further processed by selective precipitation of Al-hydroxide and Mg(OH)_2_ (Figure S11) (S2.6). The XRDs of those materials are given in Figure S12. The obtained composite Al-based materials, named c-Al(OH)_3_, and Mg(OH)_2_ were used as fire retardant fillers in b-UPR at 20, 40, and 60 wt.% addition.

#### 2.6.1. Mechanical Properties of the b-UPR/bio-Silica Composites

**Figure 8:**
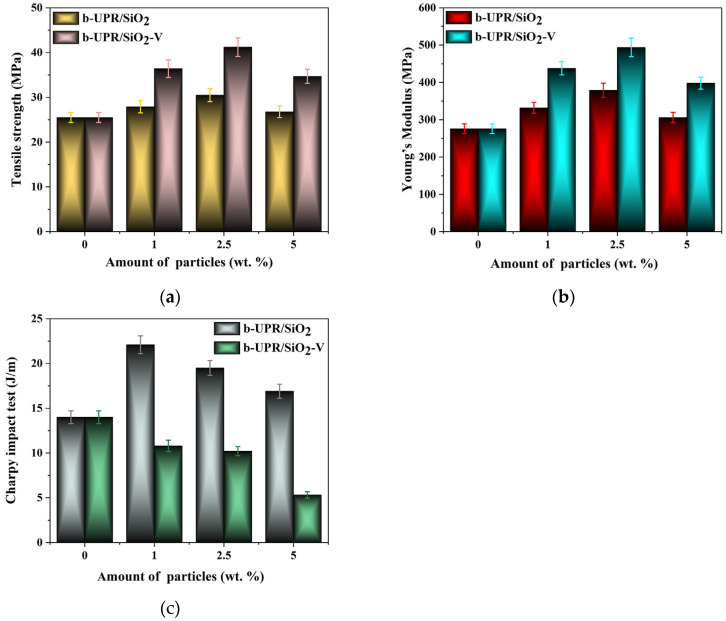
Tensile strength, Young’s modulus, and the Charpy impact strength of b-UPR/SiO_2_ and b-UPR/SiO_2_-V composites.

The highest reinforcing effect of bio-silica addition was obtained at 2.5 wt.% of both unmodified and vinyl (SiO_2_-V) modified bio-silica (S2.6.1), while the Charpy impact strength peaked at 1 wt.% of SiO_2_-V addition. The colored test specimens are given in Figure S13. The values of tensile strength, elongation, and modulus of elasticity are provided in Figure 8 and Table S15.

**Figure 9:**
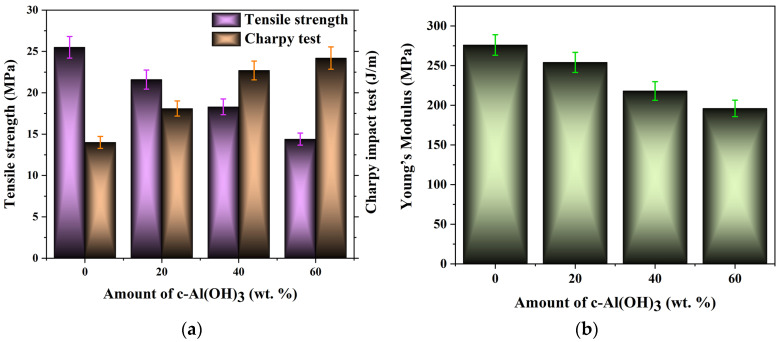
Tensile strength and Young’s modulus of b-UPR/c-Al(OH)_3_ composites.

The SEM micrograph of b-UPR/2.5 wt.% SiO_2_ composites is given in Figure S14. Moreover, the results of the mechanical properties of b-UPR/c-Al(OH)_3_ are given in Figure 9 and Table S16.

Similar results were obtained for b-UPR/Mg(OH)_2_ composites (Table S17).

#### 2.6.2. Thermal Stability of b-UPR-Based Composites

The rating of the thermal stability of the produced composites, including b-UPR/SiO_2_, b-UPR/c-Al(OH)_3_, and b-UPR/Mg(OH)_2_, was determined according to the UL-94 standard vertical test (Table 7) using the apparatus presented in Figures S15 and S18. In the flammability test, after exposing the samples to a flame [9], the dripping was more dominant during the exposure of neat b-UPR. The speed of dripping, i.e., the sample bursting, differed depending on the ratio of cross-linked b-UPR and fire-retardant addition.

When materials reach the end of their useful lives after several cycles, they can be used again as either non-reactive fillers in freshly developed UPR matrices or by evaluating the biodegradability of the composites they form.

### 2.7. Literature Survey of Adsorption Data for LDH-Based Adsorbents

The efficiency of the MgAl-LDH and MgAl-LDH@SiO_2_ particles for dyes removal are similar or higher than those of other LDH-based adsorbents (Table S18) [33,46,47,48,49,50,51,52,53,54].

## 3. Materials and Methods

### 3.1. Materials

Supplementary Material S3.1 lists all of the materials employed.

### 3.2. Syntheses of the MgAl-LDH and MgAl-LDH@SiO_2_

The methods used for the preparation of bio-silica particles and MgAl-LDH and modification of MgAl-LDH@SiO_2_ with amino-silane are given in S3.2.1, S3.2.2, and S3.2.4, respectively [55].

#### Optimization of MgAl-LDH@SiO_2_ Synthesis

The optimization goal was to produce an optimal amount of uniform coating of MgAl-LDH onto the bio-silica surface. The synthesis process was conducted using six consecutive steps [56]. The first modification step was as follows: bio-silica (100 g) was wetted with an aqueous solution of MgCl_2_ × 4H_2_O (33 mmol) and Al_2_(OH)_5_Cl × 2.5 H_2_O (11 mmol) in 30 cm^3^ deionized water and added to the reactor [56]. The test tube was filled with xylene as a non-solvent, and gentle mixing of the media was achieved by nitrogen/air bubbling in an upstream flow (for 30 min). Aqueous solutions of 1M NaOH were used to adjust the pH to 10, and the solution was left overnight. The obtained material was washed with deionized water until a neutral pH of washing was obtained and used in subsequent deposition experiment (five cycles). All materials obtained were used in the adsorption study. The optimal mass ratio of bio-silica to MgAl-LDH was ~12:1 (~7.6 wt.%) (Figure S17).

### 3.3. Synthesis of Bio-Based Unsaturated Polyester Resin (b-UPR)

The methods for the production of bio-based unsaturated polyester resin, b-UPR, as the matrix for composites preparation are given in S3.3 [57].

### 3.4. Adsorption/Desorption Study in a Batch and Fixed-Bed Column System

Details of the adsorption/desorption experiments are provided in S3.4 [58].

### 3.5. Technologies Developed for Desorbed Pollutant and Exhausted Adsorbent Disposal

To ensure environmentally friendly disposal of the effluent/treated water after desorption and exhaustion of the adsorbent, several methods for stabilizing spent adsorbent and aqueous solutions containing pollutants have been developed (S3.5).

#### 3.5.1. Disposal of Exhausted Adsorbent

After five adsorption/desorption cycles, the exhausted adsorbent was subjected to acid washing to remove the MgAl-LDH deposit, leaving b-SiO_2_ nanofiller that was used as reinforcement for biobased unsaturated polyester resin (b-UPR) (S3.5.1) [45].

#### 3.5.2. Preparation of Immobilized Enzyme on Amino-MgAl-LDH@SiO_2_ Support

After introducing amino groups onto MgAl-LDH@SiO_2_ [29], immobilization of HPR onto amino-MgAl-LDH@SiO_2_ was performed. In order to obtain a more stable immobilized preparation, amino-MgAl-LDH@SiO_2_ was activated with glutaraldehyde (GA). The protein concentration was determined using the Bradford method, and the activities of free and immobilized peroxidase were calculated. All methods, including the decolorization of dyes and the HPR recycling potential in consecutive cycles, are described in S3.5.2 [59,60,61,62,63].

#### 3.5.3. Dye Decolorization Procedure

A description of the decolorization procedure is given in S3.5.3.

#### 3.5.4. Photocatalytic Experiment

The photocatalytic protocol is presented in S3.5.4 [64,65,66,67,68].

#### 3.5.5. Disposal of Cr(VI)

The procedures for heavily soluble Cr(III)-oxide formation are given in S4.5.5 [69,70,71].

### 3.6. Characterization Methods

Comprehensive details regarding the characterization methods used are provided in S3.6 [72,73].

## 4. Conclusions

The sustainable development agenda related to the preservation of the planet is a rapidly evolving area, and a wealth of results have been achieved. Nevertheless, there are still many challenges, such as increasing the use of bio-based raw materials, decreasing energy consumption, simplifying technologies, enhancing end-of-life material recycling, and improving environmental friendliness, that should be addressed. In line with this, herein, information on the achievement of some these goals through the development of sustainable technologies for wastewater purification and the valorization of generated waste materials into useful products is presented. Three main goals were achieved:

- The production of effective MgAl-LDH and MgAl-LDH@SiO_2_ useful for anionic pollutant removal was attained. Adsorption capacities of 89.39, 275.4, 219.9, and 488.4 mg g^−1^ as well as 100.3, 307.6, 243.3, and 537.2 mg g^−1^ for Cr(VI), Acid Blue 225 (AB-225), Acid Violet 109 (AV-109), and Acid Green 40 (AG-40) dye removal using MgAl-LDH and MgAl-LDH@SiO_2_ adsorbent, respectively, were obtained.
- Effluent water obtained from desorption was successfully treated either by photocatalytic or enzymatic methods using regenerated bio-silica as support.
- The COD values decreased nearly linearly, reaching 178 mg O_2_/L after 210 min and 126 mg O_2_/L after 4 h of irradiation.
- Exhausted adsorbent MgAl-LDH@SiO_2_ was transformed to bio-silica reinforcement, and c-Al(OH)_3_ and Mg(OH)_2_ fire retardants were used in the production of b-UPR-based composites with improved mechanical and fire-proofing properties.
- The addition of 2.5% silica particles raises the composite’s tensile strength by 61.6% compared to the pure matrix. Young’s modulus exhibits a similar increasing trend, reaching 37.3% of that of the pure matrix. Adding c-Al(OH)_3_ to the polymer matrix reduces the composite’s mechanical characteristics. Tensile strength is reduced by 43.6% with the addition of 60 wt.% c-Al(OH)_3_.

At the same time, it is necessary to continue work on the development of low-cost, high-efficiency, and pollution-free technologies, promoting their widespread application in industrial production, in line with sustainable development goals.

## Figures and Tables

**Table 1:** The results of Langmuir non-linear fitting for Cr(VI) (Ci = 10 mg dm^−3^) and dyes (Ci = 25 mg dm^−3^) adsorption onto MgAl-LDH@SiO_2_.

Langmuir Model		q_m_ (mg g^−1^)	K_L_ (dm^3^ mg^−1^)	R^2^
Cr(VI)	25 °C	100.3 ± 13.5	2.31 ± 0.76	0.927
	35 °C	105.4 ± 14.9	2.41 ± 0.82	0.927
	45 °C	111.3 ± 16.9	2.56 ± 0.92	0.925
AB-225	25 °C	307.5 ± 32.5	3.11 ± 0.76	0.958
	35 °C	306.6 ± 32.3	3.51 ± 0.85	0.958
	45 °C	304.7 ± 32.4	4.03 ± 1.01	0.956
AV-109	25 °C	243.3 ± 33.1	1.69 ± 0.62	0.905
	35 °C	244.0 ± 32.0	1.86 ± 0.67	0.901
	45 °C	244.4 ± 30.9	2.08 ± 0.73	0.911
AG-40	25 °C	537.2 ± 63.8	4.18 ± 1.30	0.938
	35 °C	548.4 ± 65.4	4.49 ± 1.42	0.941
	45 °C	560.2 ± 66.9	4.90 ± 1.53	0.944

**Table 2:** Calculated thermodynamic parameters for Cr(VI), AB-225, AV-109, and AG-40 adsorption onto MgAl-LDH@SiO_2_.

Pollutant	25 °C	ΔG^Θ^ (kJ mol^−1^) 35 °C	45 °C	ΔH^Θ^ (kJ mol^−1^)	ΔS^Θ^ (J mol^−1^ K^−1^)	R^2^
Cr(VI)	−38.95	−40.37	−41.84	4.16	144.5	0.994
AB-225	−39.69	−41.32	−43.03	10.15	167.1	0.996
AV-109	−37.17	−39.71	−41.29	8.30	155.9	0.998
AG-40	−40.42	−41.96	−43.55	6.25	156.5	0.994

**Table 3:** PSO model parameters and activation energy (Ea) for the adsorption of Cr(VI), AB-225, AV-109, and AG-40 onto MgAl-LDH@SiO_2_ at 25, 35, and 45 °C.

	*T* (°C)	*q_e_* (mg g^−1^)	*k_2_* (g (mg min)^−1^)	*R^2^*	*Ea* (KJ mol^−1^)
Cr(VI)	25 °C	90.01 ± 2.61	0.00179 ± 0.0001	0.998	
	35 °C	91.60 ± 2.62	0.00202 ± 0.0002	0.998	9.13
	45 °C	93.26 ± 2.87	0.00226 ± 0.0001	0.999	
AB-225	25 °C	254.5 ± 10.4	0.00120 ± 0.0002	0.999	
	35 °C	252.8 ± 9.09	0.00150 ± 0.0001	0.999	18.4
	45 °C	251.4 ± 8.96	0.00192 ± 0.0002	0.999	
AV-109	25 °C	232.5 ± 7.86	0.00117 ± 0.0001	0.988	
	35 °C	232.9 ± 8.32	0.00126 ± 0.0001	0.998	6.18
	45 °C	233.3 ± 8.51	0.00137 ± 0.0001	0.998	
AG-40	25 °C	468.5 ± 6.15	0.00129 ± 0.0001	0.999	
	35 °C	469.9 ± 6.06	0.00134 ± 0.0001	0.999	5.61
	45 °C	470.4 ± 5.91	0.00149 ± 0.0001	0.999	

**Table 4:** Kinetic parameters of the W–M, D–W, and HSDM models for the adsorption of Cr(VI) and dyes onto MgAl-LDH@SiO_2_.

Model	Parameter	Cr(VI)	AB-225	AV-109	AG-40
W–M (Step 1)	*k*_p1_ (mg g^−1^ min^−0.5^)	9.726 ± 0.30	23.39 ± 0.94	21.07 ± 1.05	47.59 ± 1.88
	*C* (mg g^−1^)	21.81	110.9	97.47	248.23
	*R* ^2^	0.999	0.999	0.999	0.998
W–M (Step 2)	*k*_p2_ (mg g^−1^ min^−0.5^)	0.435 ± 0.01	0.264 ± 0.02	0.352 ± 0.02	0.527 ± 0.0
	*C* (mg g^−1^)	79.08	237.1	214.3	467.9
	*R* ^2^	0.998	0.998	0.998	0.998
D–W	*K*	0.0215 ± 0.00	0.0319 ± 0.00	0.0312 ± 0.00 0.0217	0.0348 ± 0.00
	*R* ^2^	0.845	0.819	0.826	0.736
HSDM	*D*s	2.48 × 10^−11^ ± 0.00	3.52 × 10^−11^ ± 0.00	3.46 × 10^−11^ ± 0.00	3.72 × 10^−11^ ± 0.00
	*R* ^2^	0.835	0.816	0.824	0.731

**Table 5:** Results of pollutant removal using MgAl-LDH@SiO_2_ (C_i_[AB-225] = C_i_[AG-40] = C_i_[AV-109] = 25 mg dm^−3^, Ci[Cr(VI)] = 10 mg dm^−3^, m_ads_ = 0.782 g, T = 25 °C, pH = 6).

Model and Parameters			*Q* (cm^3^ min^−1^)		
			0.5	1.0	1.5
B–A	*K*_BA_ (dm^3^ mg^−1^ min^−1^)	Cr(VI)	0.028 ± 6.99 × 10^−4^	0.053 ± 0.002	0.091 ± 0.003
	*q*_o_ (mg g^−1^)		90.02 ± 0.65	74.15 ± 0.93	60.83 ± 0.89
	*R* ^2^		0.999	0.997	0.997
	*K*_BA_ (dm^3^ mg^−1^ min^−1^)	AB-225	0.011 ± 4.45 × 10^−4^	0.021 ± 8.69 × 10^−4^	0.032 ± 0.00
	*q*_o_ (mg g^−1^)		294.7 ± 2.46	271.2 ± 2.84	226.6 ± 2.32
	*R* ^2^		0.992	0.990	0.993
	*K*_BA_ (dm^3^ mg^−1^ min^−1^)	AV-109	0.014 ± 3.54 × 10^−4^	0.027 ± 9.33 × 10^−4^	0.040 ± 0.001
	*q*_o_ (mg g^−1^)		233.9 ± 1.34	207.4 ± 1.85	178.8 ± 1.52
	*R* ^2^		0.997	0.995	0.997
	*K*_BA_ (dm^3^ mg^−1^ min^−1^)		0.007 ± 2.07 × 10^−4^	0.014 ± 4.34 × 10^−4^	0.020 ± 3.22 × 10^−4^
	*q*_o_ (mg g^−1^)	AG-40	555.5 ± 2.99	503.5 ± 3.23	438.0 ± 1.75
	*R* ^2^		0.992	0.992	0.998
	*k*_YN_ (min^−1^)		0.564 ± 0.01	0.533 ± 0.02	0.603 ± 0.02
	*θ* (min)	Cr(VI)	6.952 ± 0.05	5.787 ± 0.07	4.744 ± 0.07
	*R* ^2^		0.999	0.997	0.996
	*K*_YN_ (min^−1^)		0.566 ± 0.02	0.527 ± 0.02	0.529 ± 0.02
	*θ* (min)	AB-225	9.194 ± 0.08	8.377 ± 0.09	7.071 ± 0.07
	*R* ^2^		0.992	0.990	0.993
Y–N	*K*_YN_ (min^−1^)		0.688 ± 0.02	0.674 ± 0.02	0.680 ± 0.02
	*θ* (min)	AV-109	7.299 ± 0.04	6.470 ± 0.06	5.525 ± 0.05
	*R* ^2^		0.997	0.995	0.997
	*K*_YN_ (min^−1^)		0.347 ± 0.01	0.349 ± 0.01	0.332 ± 0.01
	*θ* (min)	AG-40	17.33 ± 0.09	15.55 ± 0.01	13.67 ± 0.05
	*R* ^2^		0.992	0.992	0.998

**Table 6:** The results of adsorption/desorption of the studied pollutants onto/from MgAl-LDH@SiO_2_ (C_i_ = 1 mg L^−1^, Q_des_ = 0.50 cm^3^ min^−1^; m_ads_ ~ 0.40 g) using 3.0 and 1 dm^3^ of 1 wt.% NaOH/2 wt.% NaCl for dyes and Cr(VI) desorption, respectively.

	Pollutants	Adsorption (mg g^−1^) *	Desorption (mg g^−1^) *	Desorption Efficiency (%)	*C* (mg dm^−3^) **	Δ*q*, (mg g^−1^) ***	Σ ***
I	Cr(VI)	71.3	70.1	98.2	28.1	1.3	13.5
III		63.3	61.0	96.4	24.4	2.3	
V		48.1	44.9	93.4	17.9	3.2	
I	AB-225	204.1	198.3	97.2	26.4	5.7	43.9
III		189.1	179.8	95.1	23.9	9.3	
V		168.9	157.7	93.4	21.1	11.2	
I	AV-109	189.8	181.8	95.8	24.3	7.9	48.6
III		175.5	166.5	94.9	22.2	8.9	
V		168.2	156.1	92.8	20.8	12.1	
I	AG-40	374.2	368.2	98.4	49.1	5.9	52.9
III		357.9	347.8	97.2	46.4	10.1	
V		332.4	317.2	95.4	42.3	15.3	

* Adsorbed and desorbed pollutants; ** concentration of the pollutant in effluent water; *** quantity of the irreversibly bonded pollutants in the 1st, 3rd, and 5th cycles, and overall for five cycles, respectively.

**Table 7:** Results of the flammability test UL-94V.

Sample b-UPR with Filler	First Flaming	Second Flaming	Cotton Indicator Ignited	Category
Clean b-UPR	42	59	Yes	V-2
20 wt.% c-Al(OH)_3_	18	27	No	V-1
20 wt.% Mg(OH)_2_	23	30		
40 wt.% c-Al(OH)_3_	3	6		V-0
40 wt.% Mg(OH)_2_	5	10		
60 wt.% c-Al(OH)_3_	2	3		
60 wt.% Mg(OH)_2_	2	5		

## Data Availability

The data presented in this study are available on request from the corresponding author or co-authors. The data are not publicly available.

## Supplementary Materials

The following supporting information can be downloaded at: https://www.mdpi.com/article/10.3390/ijms252111837/s1. Refs. [9,33,34,37,38,41,45,46,47,48,49,50,51,52,53,54,55,56,57,58,59,60,61,62,63,64,65,66,67,68,69,70,71,72,73] are cited in Supplementary Materials.
