# Supplementary material for: MgAl-Layered Double Hydroxide-Coated Bio-Silica as an Adsorbent for Anionic Pollutants Removal: A Case Study of the Implementation of Sustainable Technologies
Source: Int J Mol Sci. 2024 Nov 4;25(21):11837. doi: 10.3390/ijms252111837 (PMC11546314; doi:10.3390/ijms252111837)
Supplement: Supplementary file 1 [file ijms-25-11837-s001.zip › ijms-3286953-supplementary.pdf]

Supplementary material

MgAl-Layered Double Hydroxide-Coated Bio-Silica as an Adsorbent for Anionic Pollutants Removal: A Case Study of the Implementation of Sustainable Technologies

Muna Abdualatif Abduarahman[1,2], Marija M. Vuksanović[3*], Nataša Knežević[3], Katarina Banjanac[4], Milena Milošević[5], Zlate Veličković[6], Aleksandar Marinković[1]

[1] Faculty of Technology and Metallurgy, University of Belgrade, Karnegijeva 4, 11120 Belgrade, Serbia

[2] Faculty of Science, University of Sabratha, 240 Sabratha, Libya

[3] „VINČA" Institute of Nuclear Sciences - National Institute of the Republic of Serbia, University of Belgrade, Mike Petrovića Alasa 12-14, 11351 Belgrade, Serbia,

[4] Innovation Center of Faculty of Technology and Metallurgy Ltd, Karnegijeva 4, 11120 Belgrade, Serbia,

[5] Institute of Chemistry, Technology and Metallurgy - National Institute of the Republic of Serbia, University of Belgrade, Njegoševa 12, 11000 Bel, grade, Serbia,

[6] Military Academy, University of Defence, Veljka Lukića Kurjaka 33, 11000 Belgrade, Serbia,

Corresponce: marija.vuksanovic@vin.bg.ac.rs,

S2. Results and discussion

S2.1. Characterization of MgAl-LDH and MgAl-LDH@SiO$_2$ particles

S2.1.1. Morphological study

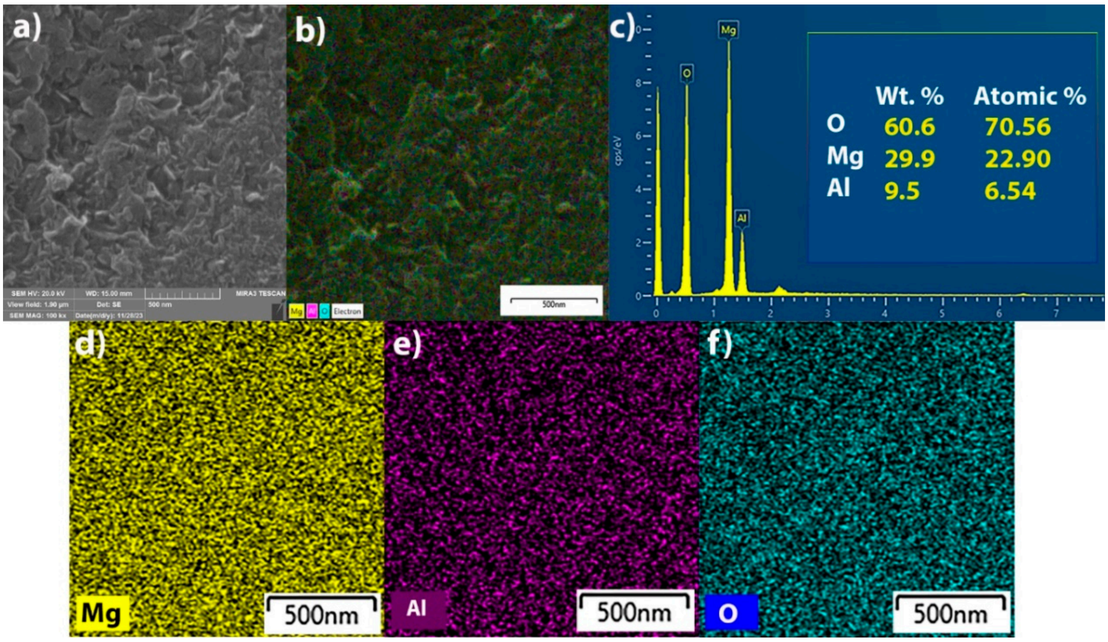

Figure S1. a) SEM images of MgAl-LDH particles, b) merged image of Mg-Al LDH, c) EDS mapping results, and d-f) elemental mapping of Mg, Al, and O.

The atomic ratio of O/Mg/Al was 70.56%/22.90%/6.54%, respectively.

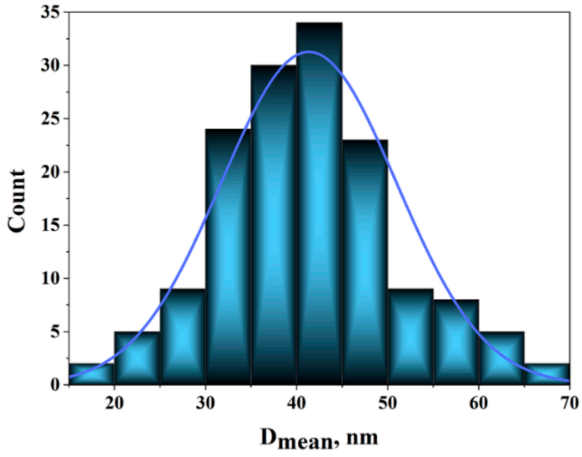

Figure S2. Diameter distribution of MgAl-LDH@SiO2 particles.

S2.1.2. FTIR structural characterization

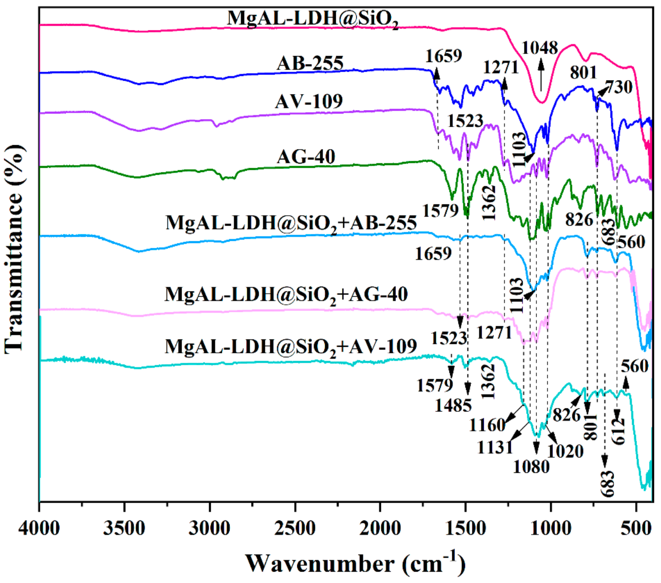

Figure S3. FTIR spectra of MgAl-LDH@SiO2 after dyes adsorption.

S2.1.3. XPS analysis

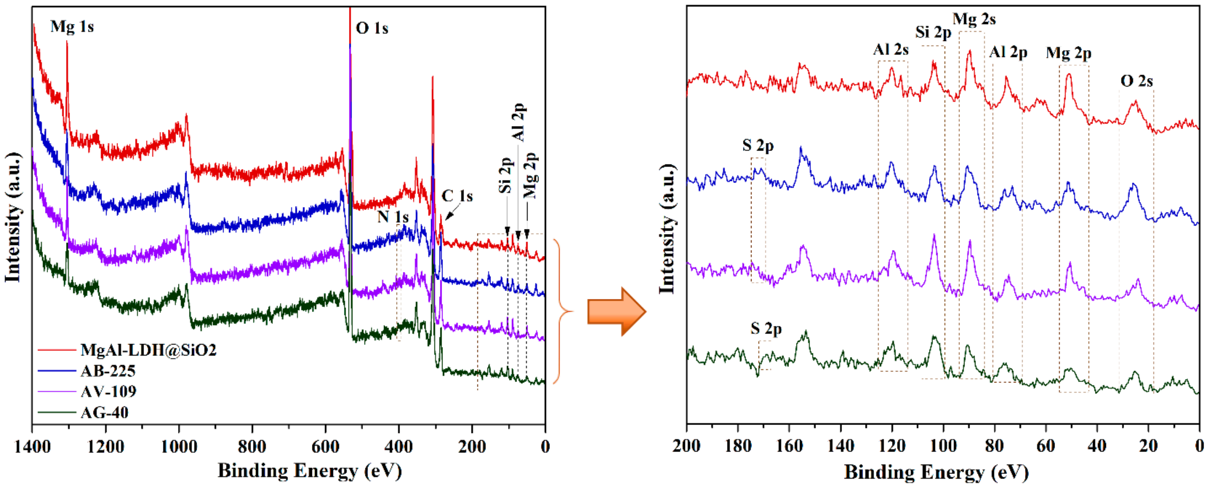

Figure S4. The survey XPS spectra of the MgAl-LDH@SiO2 before and after adsorption.

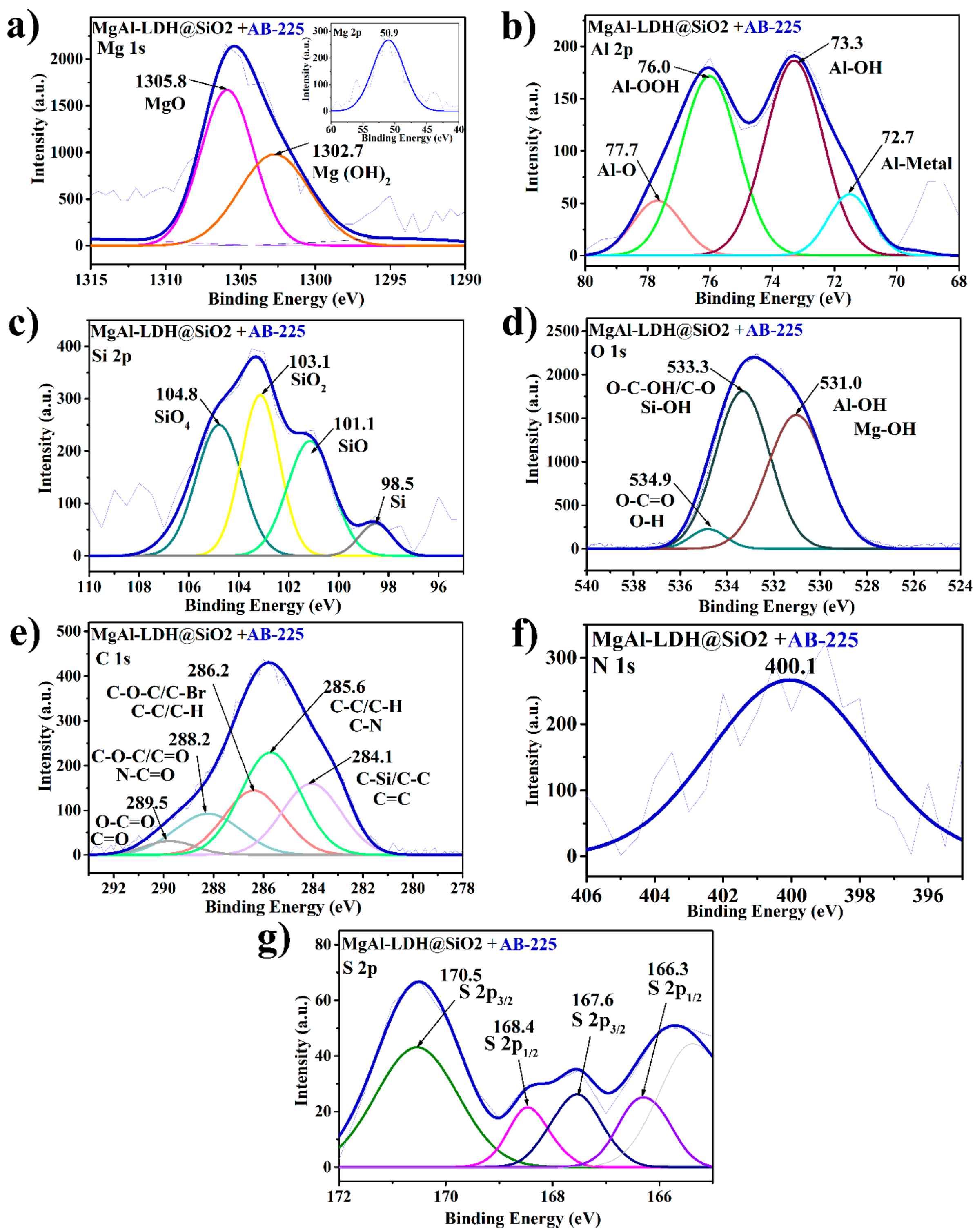

Figure S5. The core level XPS spectra of a) Mg 1s, Mg 2p, b) Al 2p, C) Si 2p, d) O 1s, e) C 1s, f) N 1s, and g) S 2p of MgAl-LDH@SiO$_2$ + AB-225.

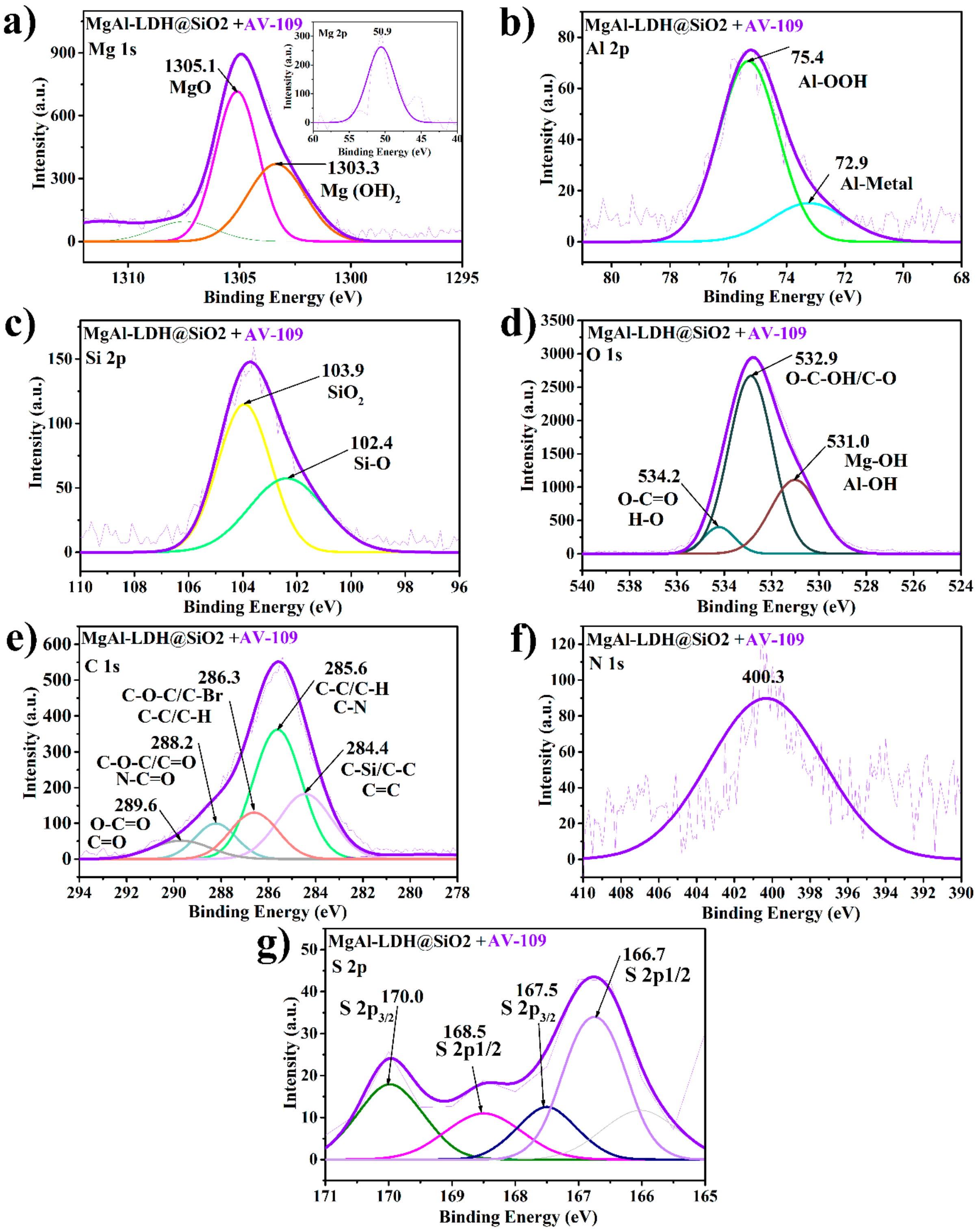

Figure S6. The core level XPS spectra of a) Mg 1s, Mg 2p, b) Al 2p, C) Si 2p, d) O 1s, e) C 1s, f) N 1s, and g) S 2p of MgAl-LDH@SiO_2 + AV-109.

S2.1.4. Determination of Zero Point Charge (pH_{PZC})

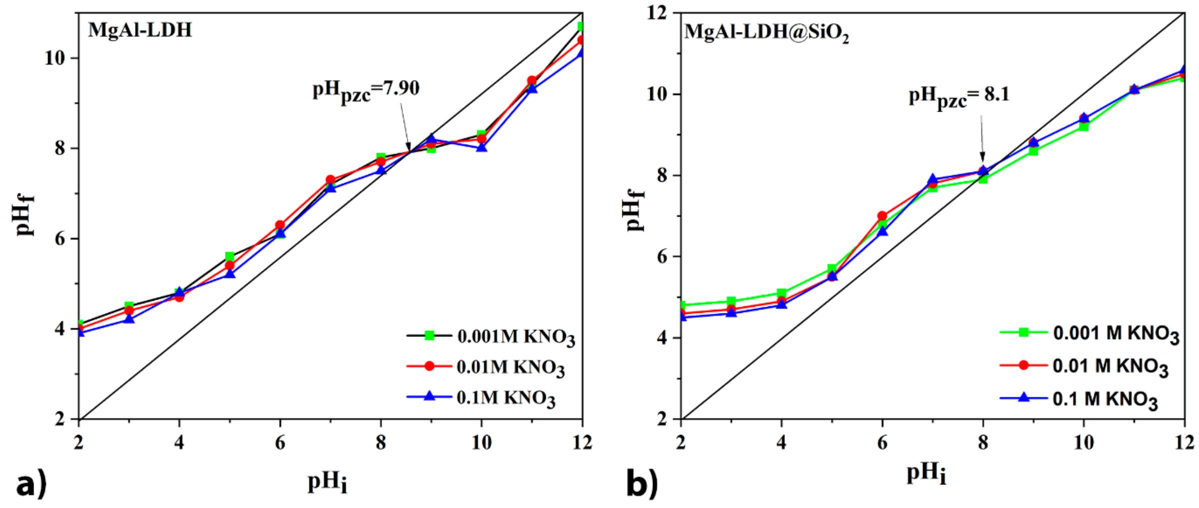

Figure S7. pHpzc of a) MgAl-LDH and b) MgAl-LDH@SiO_2.

S2.2. Adsorption studies

S2.2.1. Adsorption isotherm study

Adsorption equilibrium could be described by using different adsorption isotherm. Applying optimized adsorptive condition, the state of equilibria for both MgAl-DH and MgAl-LDH@SiO_2 was fitted using Langmuir, Eq. (S1) and Freundlich model, Eq. (S2):

$$\frac{Ce}{qe} = \frac{1}{qmKL} + \frac{Ce}{qm} \quad \text{(S1)}$$

$$log\, q\, e = log\, K\, F + \frac{1}{n} log\, C\, e \quad \text{(S2)}$$

where C_e (mg L^{-1}) and q_e (mg g^{-1}) are the equilibrium adsorbate concentration in the aqueous and solid phases. q_m (mg g^{-1}) is the maximum adsorption capacity and K_L (dm^3 mg^{-1}) is the Langmuir adsorption equilibrium constant. n is a constant indicating the Freundlich isotherm curvature, and K_F (mg g^{-1}) (dm^3 mg^{-1})$^{1/n}$ is the Freundlich equilibrium constant.

Table S1. The results of Langmuir non-linear fitting using for Cr(VI), AB-225, AV-109 and AG-40 adsorption onto MgAl-LDH particles (C_o = 10 mg L^{-1} for Cr(VI), C_o = 25 mg L^{-1} for dyes).

Langmuir model		q_m (mg g^{-1})	K_L(dm^3 mg^{-1})	R^2
Cr(VI)	25 °C	89.39 ± 8.31	2.99 ± 0.73	0.950
	35 °C	90.14 ± 8.46	3.01 ± 0.74	0.949
	45 °C	90.93 ± 8.64	3.01 ± 0.75	0.950
AB-225	25 °C	275.40 ± 29.1	12.78 ± 3.69	0.937
	35 °C	275.39 ± 28.9	13.10 ± 3.76	0.938
	45 °C	275.38 ± 28.7	13.43 ± 3.83	0.939
AV-109	25 °C	219.9 ± 23.9	2.034 ± 0.64	0.921
	35 °C	222.1 ± 24.3	2.077 ± 0.65	0.921
	45 °C	224.3 ± 24.6	2.122 ± 0.66	0.921
AG-40	25 °C	488.4 ± 37.3	4.271 ± 0.91	0.965
	35 °C	488.2 ± 34.9	4.758 ± 0.95	0.946
	45 °C	487.9 ± 32.4	5.348 ± 0.99	0.972

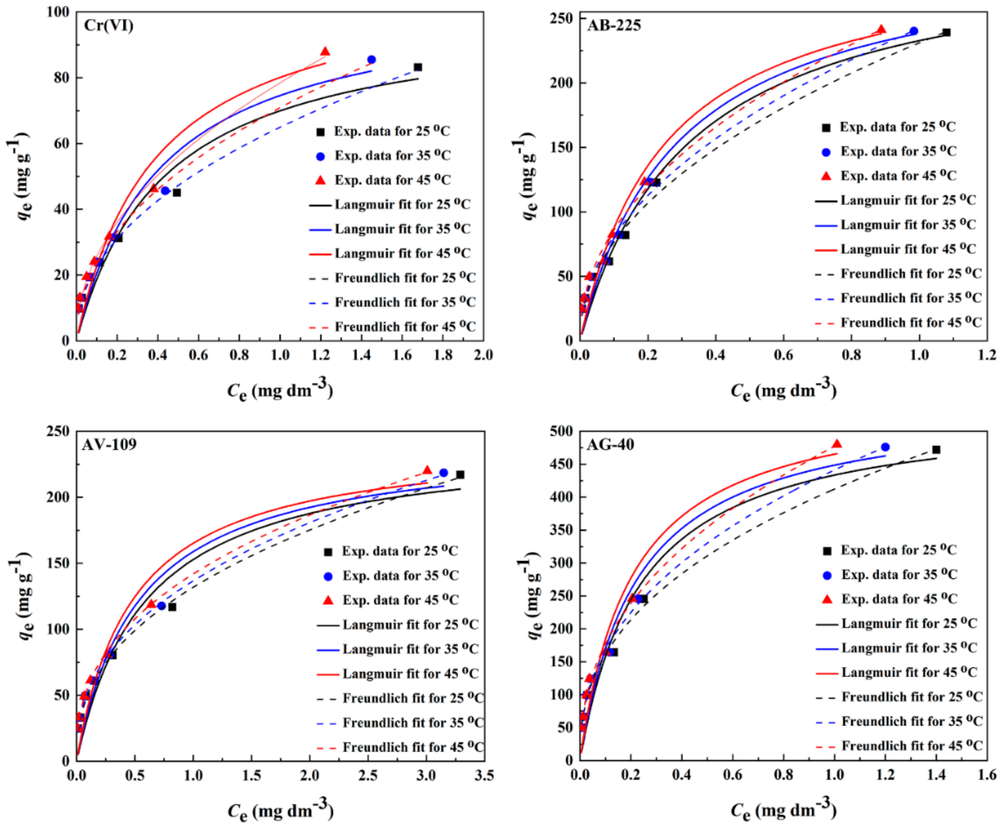

Figure S8. Adsorption isotherms for Cr(VI), AB-225, AV-109 and AG-40 adsorption onto MgAl-LDH@SiO_2 particles (C_i=10 mg L^{-1} for Cr(VI), C_i=25 mg L^{-1} for dyes).

Table S2. The results of the Freundlich isotherm model for Cr(VI), AB-225, AV-109 and AG-40 adsorption onto MgAl-LDH@SiO_2 particles (C_i = 10 mg L^{-1} for Cr(VI), C_i = 25 mg L^{-1} for dyes).

Freundlich model		K_F (mg g^{-1}) (dm^3 mg^{-1})$^{1/n}$	$1/n$	R^2
Cr(VI)	25 °C	64.96±0.78	0.458	0.997
	35 °C	70.89±1.09	0.470	0.996
	45 °C	78.45±1.58	0.483	0.994
AB-225	25 °C	231.1±6.53	0.480	0.990
	35 °C	242.5±6.59	0.476	0.992
	45 °C	255.4±6.82	0.466	0.993
AV-109	25 °C	131.8±1.53	0.411	0.998
	35 °C	136.7±1.16	0.403	0.999
	45 °C	141.9±1.05	0.394	0.999
AG-40	25 °C	412.3±7.62	0.408	0.996
	35 °C	441.3±7.41	0.418	0.997
	45 °C	477.4±7.36	0.430	0.987

Table S3. The results of the Freundlich isotherm model for Cr(VI), AB-225, AV-109 and AG-40 adsorption onto MgAl-LDH particles C_o = 10 mg L^{-1} for Cr(VI), C_o = 25 mg L^{-1} for dyes).

Freundlich model		K_F (mg g^{-1}) (dm^3 mg^{-1})$^{1/n}$	*1/n*	R^2
Cr(VI)	25 °C	60.31±0.91	0.422	0.995
	35 °C	61.07±0.91	0.424	0.996
	45 °C	61.86±0.90	0.427	0.996
AB-225	25 °C	325.6±5.77	0.400	0.998
	35 °C	328.5±5.89	0.405	0.998
	45 °C	331.4±6.05	0.404	0.998
AV-109	25 °C	122.0±1.16	0.376	0.998
	35 °C	124.2±1.13	0.378	0.998
	45 °C	126.7±1.11	0.380	0.999
AG-40	25 °C	338.5±10.3	0.362	0.987
	35 °C	347.0±11.1	0.357	0.986
	45 °C	356.0±12.1	0.352	0.985

The results of adsorption study, using MgAl-LDH@SiO_2, performed at C_i = 1 mg L^{-1} and t = 60 min for Cr(VI) and dyes are given in Table S4.

Table S4. The results of the Langmuir isotherm model for Cr(VI), AB-225, AV-109 and AG-40 adsorption onto MgAl-LDH@SiO_2 (C_i = 1 mg L^{-1}, t = 1 h).

		q_m (mg g^{-1})	K_L(dm^3 mg^{-1})	k_2 (g (mg min)$^{-1}$)
Cr(VI)	25 °C	82.35±9.05	20.49±6.56	0.001793
	35 °C	84.77±9.35	27.69±8.31	
	45 °C	87.21±10.4	39.47±12.6	
AB-225	25 °C	214.2±25.5	25.33±7.60	0.000356
	35 °C	216.9±26.2	25.93±7.79	
	45 °C	219.7±28.1	26.56±7.97	
AV-109	25 °C	198.2±23.7	20.20±6.06	0.000383
	35 °C	204.9±24.6	22.09±6.63	
	45 °C	211.5±25.4	24.28±7.28	
AG-40	25 °C	380.6±40.8	222.2±23.3	0.00031
	35 °C	387.1±41.7	258.8±28.9	
	45 °C	393.8±42.5	315.4±31.9	

S2.2.2. Thermodynamic study

Thermodynamic parameters allow estimation the feasibility of adsorption, energy change, and randomness at adsorbate/adsorbent surface. Gibbs free energy ($\Delta G^{\ominus}$), enthalpy ($\Delta H^{\ominus}$), and entropy ($\Delta S^{\ominus}$) calculated from the Van't Hoff equations, i.e., Eqs. (S3) and (S4) [1], were used as indicators of the state of the adsorption process:

$$\Delta G^{\ominus} = -RT \ln K L \quad (S3)$$

$$\ln K L = \frac{\Delta S^{\ominus}}{R} - \frac{\Delta H^{\ominus}}{RT} \quad (S4)$$

where T denotes the absolute temperature (K) and R denotes the universal gas constant (J mol^{-1} K^{-1}). Isothermal experiments yield the Langmuir adsorption constant K_L. H and S are calculated from the slopes and interceptions in the diagram ln(K_L) - T^{-1}, assuming that the system has reached stationary

conditions. The results of thermodynamic study for the system at C_i = 10 mg L^{-1} for Cr(VI) and C_i = 25 mg L^{-1} for dyes are given in Table S5.

Table S5. Calculated thermodynamic parameters for Cr(VI), AB-225, AV-109 and AG-40 adsorption onto MgAl-LDH particles.

Ion	$\Delta G^{\ominus}$ (kJ mol^{-1})			$\Delta H^{\ominus}$ (kJ mol^{-1})	$\Delta S^{\ominus}$ (J mol^{-1} K^{-1})	R^2
	25 °C	35 °C	45 °C			
Cr(VI)	-39.60	-40.93	-42.26	0.10	133.14	0.988
AB-225	-48.30	-49.98	-51.98	1.98	168.61	0.999
AV-109	-45.07	-46.63	-48.20	1.65	156.68	0.998
AG-40	-40.47	-42.11	-43.78	8.86	165.44	0.998

S2.2.3. Adsorption kinetics

The adsorption as a function of contact time was conducted at room temperature. To clarify the adsorption kinetics of Cr(VI) on LDH, pseudo-first (PFO) and pseudo-second-order (PSO) kinetic models were used. The linear form of pseudo-first-order Eq. (S5) and the pseudo-second-order Eq. (S6) can be expressed as follows:

$$ln(q_e - q_t) = ln\, q_e - k_1 t \quad (S5)$$

$$q_t = \frac{t}{\left(\frac{1}{k2qe^2} + \frac{1}{qe}\right)} \quad (S6)$$

where q_e and q_t (mg/g) are the amounts of arsenic adsorbed on the adsorbents at equilibrium and at time t (min). k_1 (min^{-1}) and k_2 (g mg^{-1} min^{-1}) are the rates constant of pseudo-first-order and pseudo-second-order kinetic models.

The Weber-Morris, Dunwald-Wagner, and HSDM kinetic models are given by Eqs. (S7– S9) [2]:

$$qt = kit^{\frac{1}{2}} + C \quad (S7)$$

$$K_{DW} = \pi^2 D_{DW} R^2 \quad (S8)$$

$$\frac{qt}{qe} = 1 + \frac{2R}{\pi r}\sum_{n=1}^{\infty}\frac{(-1)^n}{n} sin\frac{nr\pi}{R} exp\left[\frac{-DSt\pi^2 n^2}{R^2}\right] \quad (S9)$$

Where k_i (mg g^{-1} $min^{-1/2}$) is the intraparticle diffusion rate constant and C (mg g^{-1}) is a constant which reflects the boundary layer effect. K_{DW} (min^{-1}) is the rate constant of adsorption while D_{DW} (m^2 min^{-1}) is the diffusion coefficient of the migrating species, r is the initial radius of the reacting particle (m), q_e and q_t are the adsorption capacity of adsorbent (mg g^{-1}) at equilibrium and appropriate t, respectively. Ds is the intraparticle diffusion coefficient, r radial position, and q *is* the time-dependent adsorption capacity.

Table S6. PSO model parameters and activation energy (E_a) for the adsorption of Cr(VI), AB-225, AV-109 and AG-40 onto MgAl-LDH at 25, 35, and 45 °C.

	T, °C	q_e (mg g^{-1})	k_2 (g (mg min)$^{-1}$)	R^2	E_a (KJ/mol)
Cr(VI)	25	86.81±2.61	0.00178 ± 0.0001	0.997	5.25
	35	86.92±2.62	0.00190 ± 0.0002	0.998	
	45	87.04±2.87	0.00204 ± 0.0001	0.998	
AB-225	25	259.9±7.59	0.00110 ± 0.0002	0.933	4.41
	35	259.7±6.49	0.00117 ± 0.0002	0.946	
	45	259.6±5.46	0.00123 ± 0.0002	0.958	
AV-109	25	228.9±6.03	0.00091 ± 0.0001	0.968	4.58
	35	229.1±5.92	0.00096 ± 0.0001	0.966	
	45	229.4±5.76	0.00102 ± 0.0001	0.964	

	25	474.8±6.17	0.00107 ± 0.0001	0.965	
AG-40	35	475.5±6.19	0.00113 ± 0.0001	0.962	4.09
	45	476.2±6.22	0.00119 ± 0.0001	0.958	

Table S7. Kinetic parameters of the W-M, D-W, and HSDM models for the adsorption of Cr(VI), AB-225, AV-109 and AG-40 onto MgAl-LDH.

Model	Constants	Cr(VI)	AB-225	AV-109	AG-40
W-M (Step 1)	k_{p1} (mg g^{-1} min$^{-0.5}$)	8.612	26.46±0.94	27.61± 0.05	45.22±1.88
	C (mg g^{-1})	23.02	103.6	60.83	242.6
	R^2	0.999	0.998	0.999	0.994
W-M (Step 2)	k_{p2} (mg g^{-1} min$^{-0.5}$)	0.286 ± [illegible]	0.439± 0.016	0.484±0.027	0.703±0.033
	C (mg g^{-1})	77.33	241.6	204.3	447.7
	R^2	0.998	0.999	0.999	0.998
D-W	K_{DW}	0.0214	0.0316±0.001	0.0292±0.0012 [illegible]	0.035±0.0042
	R^2	0.844	0.821	0.822	0.748
HSDM	D_s	2.47E-[illegible]	3.50E^{-11}	3.32E^{-11}	3.74E^{-11}
	R^2	0.837	0.817	0.815	0.741

S2.2.4. Continuous-flow experiments

Table S8. Results of the pollutants removal onto MgAl-LDH (C_i[AB-225] = C_i[AG-40] = C_i[AV-109]=25 mg dm^{-3}, C_i[Cr(VI)]=10 mg dm^{-3}, m_{ads} = 0.754 g, T = 25 °C, pH = 6)

Model and parameters			Q (cm^3 min^{-1})		
			0.5	1.0	1.5
B-A	K_{BA} (dm^3 mg^{-1} min^{-1})	Cr(VI)	0.0571± 0.002	0.0752 ± 0.002	0.0905± 0.003
	q_o (mg g^{-1})		73.67± 0.53	70.71± 0.59	63.01± 0.93
	R^2		0.998	0.998	0.996
	K_{BA} (dm^3 mg^{-1} min^{-1})	AB-225	0.014±3.23E-4	0.027±8.40E-4	0.043± 0.001
	q_o (mg g^{-1})		244.7 ±1.23	211.7±1.73	161.6±1.32
	R^2		0.998	0.996	0.997
	K_{BA} (dm^3 mg^{-1} min^{-1})	AV-109	0.0158± 3.63E-4	0.0330± 7.31E-4	0.0492±0.001
	q_o (mg g^{-1})		204.1±1.07	182.9±1.00	165.4±1.30
	R^2		0.998	0.998	0.997
	K_{BA} (dm^3 mg^{-1} min^{-1})	AG-40	0.007± 1.49E-4	0.015± 2.31E-4	0.022± 2.92E-4
	q_o (mg g^{-1})		495.6±2.24	432.4±1.53	351.0±1.38
	R^2		0.996	0.998	0.999
Y–N	K_{YN} (min^{-1})	Cr(VI)	1.142± 0.05	0.752± 0.02	0.603± 0.02
	θ (min)		5.555± 0.04	5.331± 0.04	4.751± 0.07
	R^2		0.998	0.998	0.996
	K_{YN} (min^{-1})	AB-225	0.070± 0.016	0.672± 0.021	0.727± 0.019
	θ (min)		7.306± 0.04	6.385±0.05	4.826± 0.04
	R^2		0.998	0.996	0.997
	K_{YN} (min^{-1})	AV-109	0.793± 0.02	0.824± 0.02	0.821± 0.02
	θ (min)		6.154± 0.03	5.516± 0.03	4.989± 0.04
	R^2		0.998	0.998	0.997

K_{YN} (min^{-1})		0.352± 0.01	0.375± 0.01	0.362± 0.005
θ (min)	AG-40	14.80± 0.07	13.04± 0.05	10.59± 0.041
R^2		0.996	0.998	0.999

Column studies were carried out to examine the adsorbent capacity for large sample volumes. MgAl-LDH@SiO_2 adsorbent was packed in a column with a diameter of 0.7 cm, and the solution of textile dyes = 25 mg dm^{-3} was passed through the column at a flow rate of 0.5 and 1.0 cm^3 min^{-1}. The volume corresponding to 10 % of inlet concentration is termed breakthrough volume, while the volume corresponding to 90 % is termed exhaustion volume. The breakthrough time (BT) was designated as the feed time supplied with a column up to the maximum permissible limit (MPL) pollutant. According to the following equations (S10-S12) of breakthrough capacity, exhaustion capacity, and column utilization were calculated.

$$\text{Breakthrough capacity}(mg/g) = \frac{\text{Breakthrough volume}(dm^3)\times\text{inlet concentration}(mg/dm^3)}{\text{adsorbent weight }(g)} \quad \text{(S10)}$$

$$\text{Exhaustion capacity}(mg/g) = \frac{\text{Exhaustion volume}(dm^3)\times\text{inlet concentration}(mg/dm^3)}{\text{weight of the adsorbent}(g)} \quad \text{(S11)}$$

$$\text{Degree of column utilization}(\%) = [\text{Exhaustion volume}(dm^3) \times \text{Breakthrough volume}(dm^3)] \times 100$$

(S12)

Table S9. Column parameters.

Column parameters	AB-225			AG-40			AV-109		
Flow rate (cm^3 min^{-1})	0.5	1.0	1.5	0.5	1.0	1.5	0.5	1.0	1.5
Initial adsorbate concentration (mg dm^{-3})	25	25	25	25	25	25	25	25	25
Breakthrough volume (dm^3)	5.0	4.0	3.0	10.0	8.5	7.0	4.0	3.0	2.0
Exhaustion volume (dm^3)	12.5	11.5	11.0	22.0	20.5	20.0	10.0	9.0	8.5
Breakthrough time (min)	16000	7500	3666	31000	13500	8000	12000	5500	3333
Breakthrough capacity (mg g^{-1})	172.2	139.5	108.1	328.1	278.7	230.2	139.7	107.4	76.6
Exhaustion capacity (mg g^{-1})	296.6	269.2	231.8	548.8	492.3	436.9	237.8	209.0	180.6
Breakthrough time capacity (mg g^{-1})	245.2	222.5	169.3	466.6	404.1	342.4	190.2	168.2	137.9
Degree of column utilization (%)	40.0	34.7	27.3	45.4	41.5	35.0	40.0	33.3	23.5

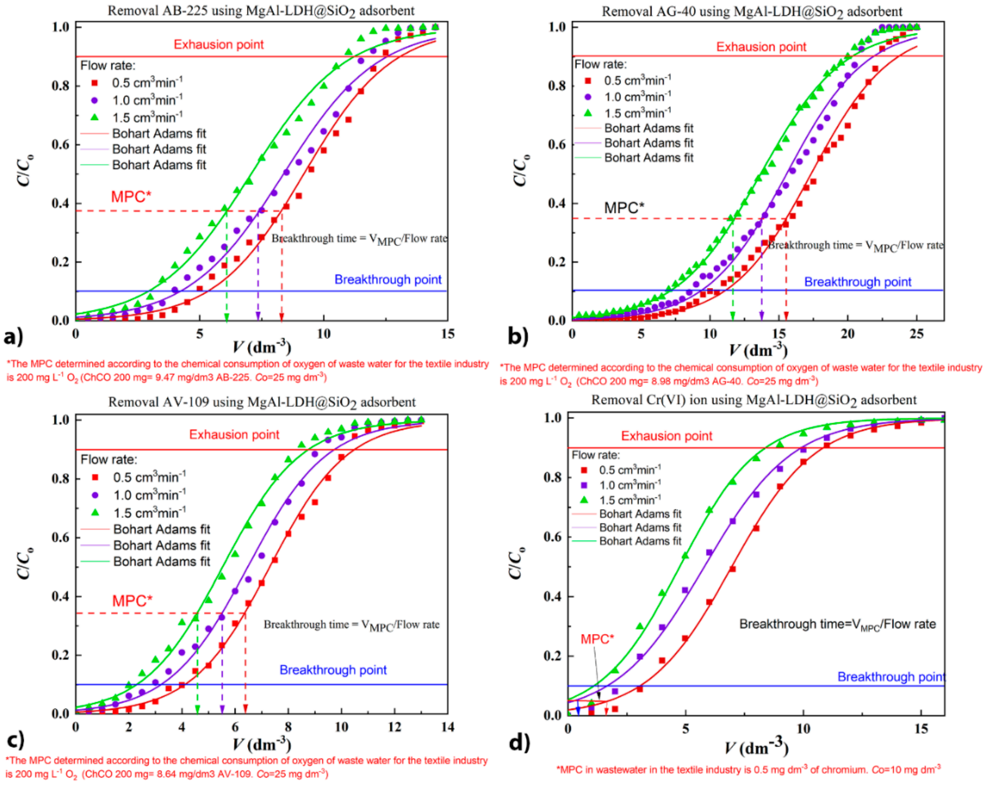

Figure S9. Bohart-Adams breakdown curves for adsorption of (a) AB-225, (b) AG-40, (c) AV-109), and (d) Cr(VI) at different flow rate onto MgAl-LDH@SiO_2.

S2.3. Desorption study in a column system

The results of desorption study at feed water concentration (25 mg dm^{-3}) and low volume of desorption solution (200 mL) are given in Table S10. The influence of inlet water flow rate is given for AB-225 dye. The optimal flow rate was found to be 0.5 cm^3 min^{-1}.

Table S10. The results of adsorption/desorption of the AB-225, AG-40, AV-109 and Cr(VI) onto/from MgAl-LDH@SiO_2 (C_i = 25 mg dm^{-3}, Q_{des} = 0.50 for all dyes and Cr(VI), and 1.00 cm^3 min^{-1} for AG-40; m_{ads} = 0.41 g) using 200 mL of 2 wt.% NaOH/2 wt.% NaCl.

Cycle	Pollutants	Adsorption (mg g^{-1})*	Flow rate (cm^3 min^{-1})	Desorption mg/g	Desorption efficiency (%)	C (mg dm^{-3})**	Δq (mg g^{-1})***	Σ***
I	AB-225	294.7	0.5	277.6	94.2	555.2	17.1	43.2
III		237.8	0.5	207.1	87.1	414.2	30.7	
V		183.3	0.5	148.6	81.1	297.3	34.6	
I	AG-40	555.5	0.5	529.9	95.4	1059.9	25.5	234.4
III		485.8	0.5	434.8	89.5	826.6	51.1	
V		377.8	0.5	316.2	83.7	548.1	53.4	
I		555.5	1	517.7	93.2	1035.5	37.8	292.6
III		462.7	1	396.5	85.7	793.1	66.2	
V		322.8	1	257.6	79.8	515.2	65.2	

I	AV-109	234	0.5	219.9	94.1	439.9	14.0	114.7
III		197.5	0.5	173.6	87.9	347.2	23.9	
V		146.9	0.5	115.9	78.9	231.8	30.9	
I	Cr(VI)	90	0.5	80.6	89.5	161.1	9.5	41.1
III		68.7	0.5	57.3	83.4	114.6	11.4	
V		48.3	0.5	38.5	79.8	77.1	9.6	

* Adsorption capacity and quantity of desorbed pollutants; ** concentration of the pollutant in effluent water; ***quantity of the irreversibly bonded pollutants.

The disposal technologies of the dye's solution obtained at low C_i is presented in Subsections 2.4 and 2.5 of the main text, which provided an effective way of the decrease of harmful effect of present pollutants in effluent water.

Otherwise, the development of the technology for disposal of dye's solution at high effluent concentration (Table S10) did not offer number of alternatives. The most valuable one was developed in following (given at example for AG-40): the dye solution (~810 mg dm^{-3}) was treated with Al-sulphate (approx. 3:1.1 molar ratio) for 10 min under ultrasound and 3 h under mixing. Al-sulphate was produced by reaction of c-$Al(OH)_3$ and sulfuric acid (2:3 molar ratio). After precipitation of the Al-salt of AG-40 dye the solution contain less than 1 mg dm^{-3} residual dye (the determined COD value satisfy national legislation, subchapter S4.5.4.). Precipitate, Al-salt of AG-40 dye, was re-dispersed in deionized water, treated with NaOH solution (1 mol dm^3) to provide pH 6.5. After filtration, i.e. separation of Al-hydroxide, the concentrated dye solution was evaporated and used for coloration of UPR based composites (Figure S13). Similar coloration was obtained using Al-salt of AG-40 dye.

S2.4.2. Activation of support with glutaraldehyde

Table S11. Characteristics of immobilized enzyme preparations.

Support	Protein loading (mg/g of support)	Activity of immobilized enzyme (IU/g of support)	Specific activities
Amino-MgAl-LDH@SiO_2	5	2158	431.6
GA-Amino-MgAl-LDH@SiO_2	3.6	1695	470.8

S2.4.3. Decolorization efficiency and Reusability study

Peroxidase requires peroxide as a mediator for colour degradation, so the optimal concentration was determined and used in further experiments.

Table S12. Influence of hydrogen peroxide concentration on the decolorization of AV-109 dye.

Concentration of H_2O_2 (%)	Decolorization degree (%)
0.05	29.3
0.08	59.5
0.12	41.9

From Table S12, it is evident that the catalytic yield is favourable at higher hydrogen peroxide concentrations. Increasing the H_2O_2 concentration above 0.08% induced a slight decrease in decolorization efficiency (about 59%). This phenomenon can be explained by the fact that an excess of hydrogen peroxide leads to a higher generation of intermediate species that inhibit enzyme activity [3]. The optimal dose for decolorization dyes using immobilized HRP was 0.08%.

S2.5. Photodegradation of effluent water containing AG-40

Table S13. COD values of initial and photo-catalytically treated solution of AG-40.

AG-40 samples*	COD (mg O_2/L)
$AG\text{-}40_0$	576
$AG\text{-}40_{1/2}$	326
$AG\text{-}40_1$	178

* A-40 solution at different reaction time: $AG\text{-}40_0$ t = 0, $AG\text{-}40_{1/2}$ *t* = 105 min, $AG\text{-}40_1$ *t* = 210 min

Also, treatment of AG-40 enriched water under photolysis was of limited successfulness: 568 mg O_2/L after 2 h, and 562 mg O_2/L after 4 h, which confirm that natural condition will not provide short term efficient degradation, but prolonged treatment period (12 days) provide COD of 270 mg O_2/L which is away from the maximum allowable value prescribed by national authority. In that way, it was proved that applied technology showed indicative results to be applied in a realistic system for wastewater purification and met the current ecological criteria to be released into watercourses. Additional work will be devoted to ecotoxicological study to confirm non-harmful properties of effluent water.

Quantum yield determination

The quantum yields (Φ) determination for photocatalytic process at example of AG-40 dye degradation are 0.330 (5 mg dm^{-3}) and 0.162 (10 mg dm^{-3}, pH 6 and T = 25 °C). Possible interactions/interference of different anions with generated structural fragments of AG-40 from the parent molecule could cause alterations increase/decrease of degradation rate. Thus, the effect of common anions on change of photodegradation rate was studied with an aim of better understanding of the change of photodegradation rate in real condition [4]. The results in Table S14 show that photodecomposition of AG-40 takes place at a higher rate than in the presence of added ions.

Table S14. Calculated values of Φ for photocatalytic processes using ZnO (pH 6, *t* = 25 °C).

Anions/catalyst	ZnO
Cl^-	0.142
NO_3^-	0.158
SO_4^{2-}	0.147
CO_3^{2-}	0.171
HPO_4^{2-}	0.018
HCO_3^-	0.066

S2.6. Recycling of exhausted MgAl-LDH@SiO2

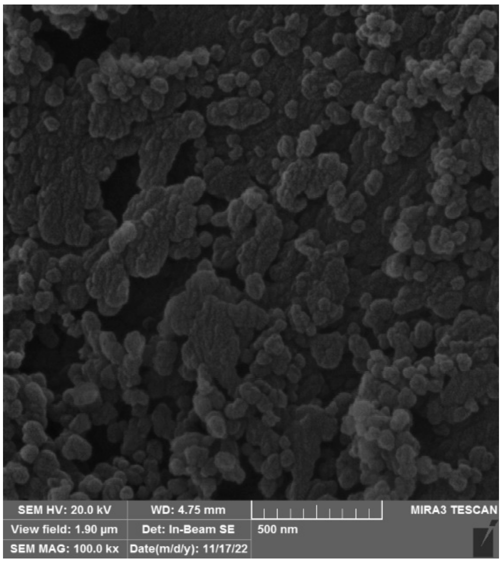

Figure S10. SEM micrograph of recycled nano-silica particles.

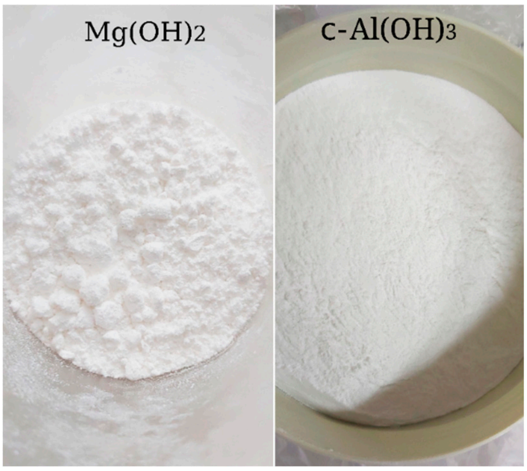

Figure S11. Powder of $Mg(OH)_2$ and c-$Al(OH)_3$.

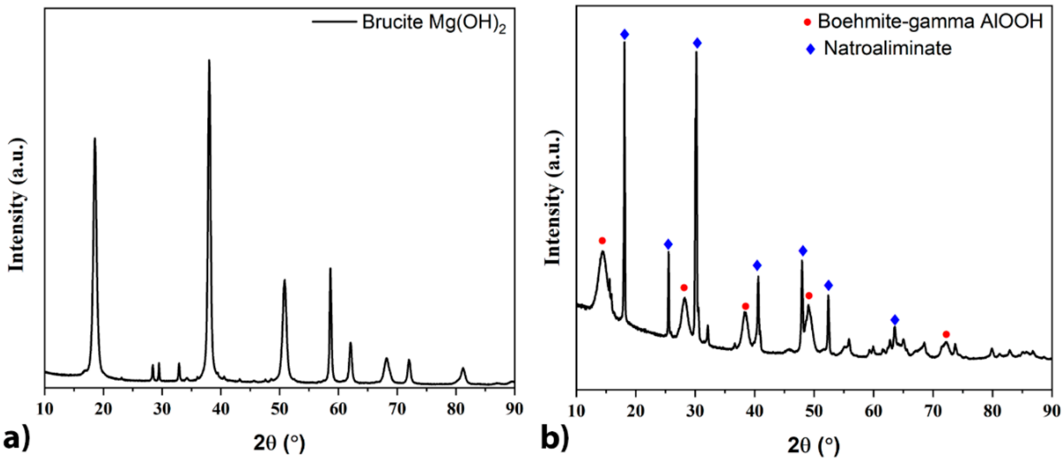

Figure S12. XRD of a) $Mg(OH)_2$ particles and b) heterogeneous material containing boehmite (γ-AlOOH) and natroaluminate $Al(OH)_3$ (named c-$Al(OH)_3$).

S2.6.1. Mechanical properties of the b-UPR based composites

b-UPR was used as a polymer matrix, and bio-silica particles were used as composites reinforcement. Composites based on b-UPR and either unmodified or modified bio-silica particles were subjected to homogenization procedure to obtain uniform nano-filler dispersion. The content of the reinforcement used in the preparation of composites are 1, 2.5 and 5 wt. %. Bio-silica particles were dispersed in b-UPR using a laboratory planetary mixer adopted to vacuum evacuation of gaseous bubbles from processing material (10 min). After that period, the initiator MEKP (1 wt. %) and 0.5 wt. % cobalt-octoate was added and homogenization (200 o/min) for 2 min under vacuum was performed to obtain homogeneous pasty dispersion which was immediately poured into moulds. Curing of composite at ambient temperature for 24 h was followed by heating at 80 °C for 4 h. In an analogous manner were prepared composite using 20, 40 and 60 wt.% addition of c-Al$(OH)_3$ and $Mg(OH)_2$, and b-UPR/c-Al$(OH)_3$(40wt.%)/b-SiO_2 and b-UPR/c-Al$(OH)_3$ (40wt.%)/b-SiO_2 (vinyl modified).

The specimen of b-UPR/2.5 wt. % SiO_2 coloured with dyes isolated from desorption solution (subchapter S3.3 Desorption study in a column system) are given in Figure S16. Mechanical properties vary in the range ±1.2% with respect to test specimen produced without colouring (Table S17).

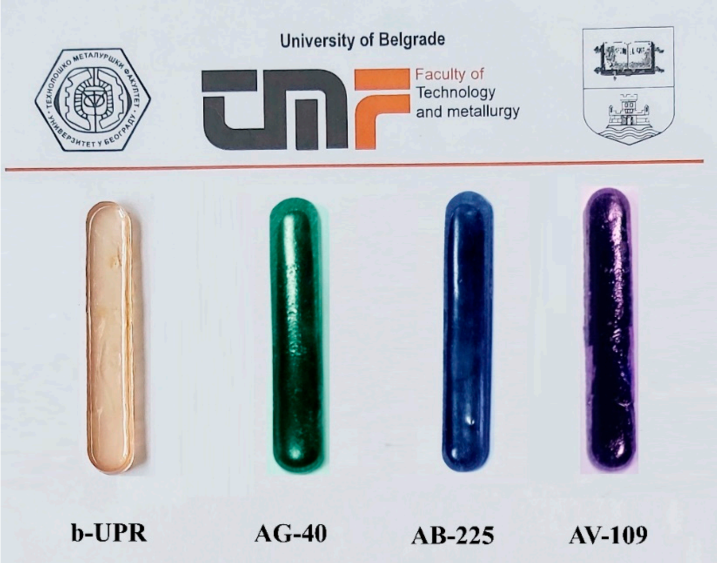

Figure S13. The specimen of b-UPR/SiO_2 coloured with dyes isolated from desorption solution.

Table S15. Mechanical properties of b-UPR/SiO_2 (recycled) composites.

Sample	σ (MPa)	ε (%)	E (MPa)	*Charpy* (J/m)
b-UPR [5]	25.5±1.11	5.90 ± 0.25	276 ± 13.0	14.0 ± 0.72
b-UPR/1 wt. % SiO_2	27.9 ± 1.39	5.74 ± 0.29	332 ± 14.3	22.1 ± 0.99
b-UPR/2.5 wt. % SiO_2	30.5 ± 1.43	5.51 ± 0.22	379 ± 19.0	19.5 ± 0.83
b-UPR/5 wt. % SiO_2	26.8 ±1.31	5.86 ± 0.24	306 ± 13.8	16.9 ± 0.79
b-UPR/1 wt.% SiO_2-V	36.4 ± 1.97	4.77 ± 0.21	438 ± 17.5	10.8 ± 0.64
b-UPR/2.5 wt.% SiO_2-V	41.2 ± 2.06	4.42 ± 0.22	494 ± 24.7	10.2 ± 0.51
b-UPR/5 wt.% SiO_2-V	34.7 ± 1.57	4.93 ± 0.28	398 ± 15.9	5.32 ± 0.36

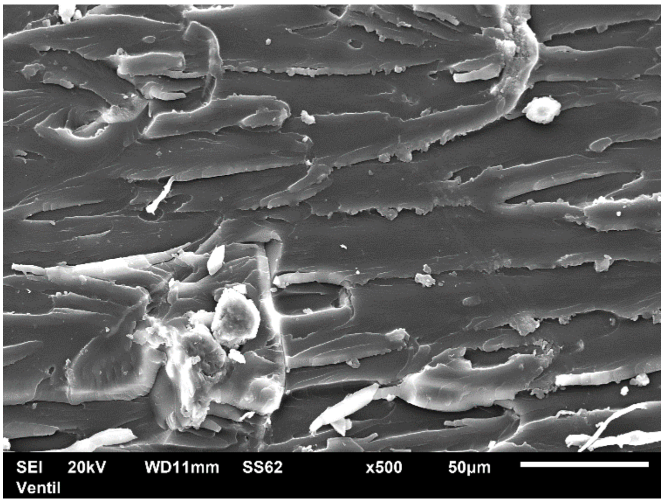

Figure S14. SEM micrograph of b-UPR/2.5 wt. % SiO_2 composites.

Table S16. Mechanical properties of b-UPR/c-$Al(OH)_3$ (recycled) composites.

Sample	*σ* (MPa)	*ε* (%)	*E* (MPa)	*Charpy* (J/m)
b-UPR [5]	25.5 ± 1.31	5.90 ± 0.25	276 ± 13.0	14.0 ± 0.72
b-UPR/20 wt. % c-$Al(OH)_3$	21.6 ± 1.15	6.12 ± 0.31	254 ± 12.7	18.1 ± 0.92
b-UPR/40 wt. % c-$Al(OH)_3$	18.3 ± 0.95	6.52 ±0.35	218 ± 11.8	22.7 ± 1.13
b-UPR/60 wt. % c-$Al(OH)_3$	14.4 ± 0.73	6.89 ± 0.41	196 ± 10.3	24.2 ± 1.35

Table S17. Mechanical properties of b-UPR/$Mg(OH)_2$ (recycled) composites.

Sample	*σ* (MPa)	*ε* (%)	*E* (MPa)	*Charpy* (J/m)
b-UPR [5]	26.1 ± 1.35	5.92 ± 0.27	278 ± 13.2	14.3 ± 0.72
b-UPR/20 wt. % $Mg(OH)_2$	22.3 ± 1.24	6.14 ± 0.29	257 ± 12.9	18.6 ± 0.92
b-UPR/40 wt. % $Mg(OH)_2$	18.6 ± 1.12	6.57 ±0.31	222 ± 12.2	23.1 ± 1.13
b-UPR/60 wt. % $Mg(OH)_2$	14.7 ± 0.89	6.90 ± 0.45	198 ± 10.5	24.4 ± 1.35

S2.6.2. Thermal stability of b-UPR based composites

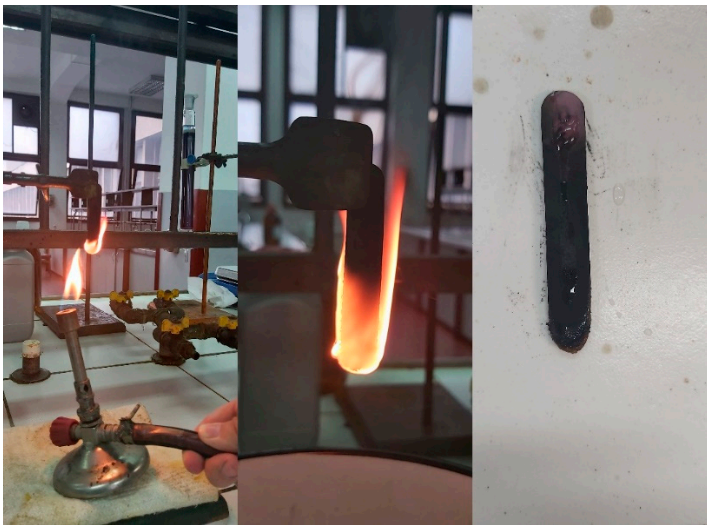

Figure S15. The apparatus set up for the vertical thermal stability test.

S2.7. Literature survey of the adsorption data for LDH base adsorbents

Table S18. A comparative overview of the adsorption capacities of LDH as adsorbents.

Adsorbent	Pollutant	C_i (mg dm^{-3})	q_m (mg g^{-1})	Reference
3D-MgAl-LDH	Acid Orange-7	600	485.6	[6]
	Methylene Blue	100	58.3	
MgAl-LDH	Acid orange 10	500	421.5	[7]
Mn-Fe_LDH/PES	Malachite green	4-12	13.5	[8]
Mg/Al–LDH	Congo red	200	520.8	
Fe_3O_4@MgAl–LDH	Congo red	200	813	[9]
ZnAl-LDH	Cr(VI)	40	118	[10]
MgAlLDH/ PBC	Cr(VI)	10–250	178	[11]
PDOPA-f-LDH	Methylene Blue	50	132	[12]
PDA/MgAl-LDH	Cr(VI)	10-200	87.02	[13]
MMA-80	Methyl Orange	60-200	440.8	[14]
	Methylene Blue	100-350	866.8	
LDH-4	Cr(VI)	50-200	25.47	[15]
C-LDH-4			33.69	
MgAl-LDH	Cr(VI)	10	89.39	This work
	AB-225	25	275.4	
	AV-109	25	219.9	
	AG-40	25	488.4	
MgAl-LDH@SiO_2	Cr(VI)	10	100.3	This work
	AB-225	25	307.6	
	AV-109	25	243.3	
	AG-40	25	537.2	

S3.1. Materials

Material used for MgAl-LDH synthesis: $MgCl_2 \times 4H_2O$ and $Al_2(OH)_5Cl \times 2.5H_2O$ (Locron L, Clariant). Sodium hydroxide (NaOH), potassium hydroxide (KOH), acetic acid, hydrochloride acid (HCl), dimethylformamide (DMF), sulfuric acid (H_2SO_4), ethanol 96 %, toluene, potassium nitrate (KNO_3), (3-aminopropyl) trimethoxysilane (amino-silane), potassium ferrioxalate $K_3[Fe(C_2O_4)_3]$, glutaraldehyde (GA), hydrogen peroxide (H_2O_2), all p.a. quality, were provided by Sigma Aldrich, Germany. Xylene, and chromium standards 1000 g/mL, were supplied by Merck, Germany.

Styrene, tetrabutyl titanate (TBT), cobalt octoate (Co-oct), hydroquinone (HQ), 5-[(formyloxy)methyl] furfural (FMF), copper (II) nitrate trihydrate ($Cu(NO_3)_2 \cdot 3H_2O$), Manganese(IV) oxide (MnO_2), and methyl ethyl ketone peroxide (MEKP) were supplied by Sigma Aldrich, Darmstadt, Germany. Fascat 4100 (butylstannoic acid,) was supplied by PMC Organometallix, Netherlands. To produce unsaturated polyester resin, waste PET collected from soft drink bottles was used. The waste PET bottles were cut into small pieces (approximately 0.5 × 0.5 cm) and washed with detergent and ethanol to remove all traces of impurities and residual adhesives.

Horseradish peroxidase (HRP) (EC 1.11.1.7; donor: hydrogen peroxide oxidoreductase) with a specific activity of 250 IU/mg was purchased from Sigma-Aldrich.

Anthraquinone dye 5-((1-amino-4-((3-(2,3-dibromopropanamido)-2,4,6-trimethylphenyl)amino)-9,10-dioxo-9,10-dihydroanthracen-2-yl)oxy)-2-(tert-butyl)benzene- sulfonate (C.I. Acid Violet 109 (AV-109)), sodium (Z)-1-amino-4-((3-(2,3-dibromo-acrylamido)-2,4,6-trimethylphenyl)amino)-9,10-dioxo-9,10-dihydroanthracene-2-sulfonate (C.I. Acid Blue 225 (AB-225)) and monosodium mono(5,5'-((((9,10-dioxo-9,10-dihydroanthracene-1,4-diyl)bis(azanediyl))bis(4,1-phenylene))bis(oxy))bis(2-chlorobenzene-sulfonate)) (C.I. Acid Green 40 (AG-40)) were provided by Lanaset (Bayer AG, Germany). The structure of dyes is given in Figure S16.

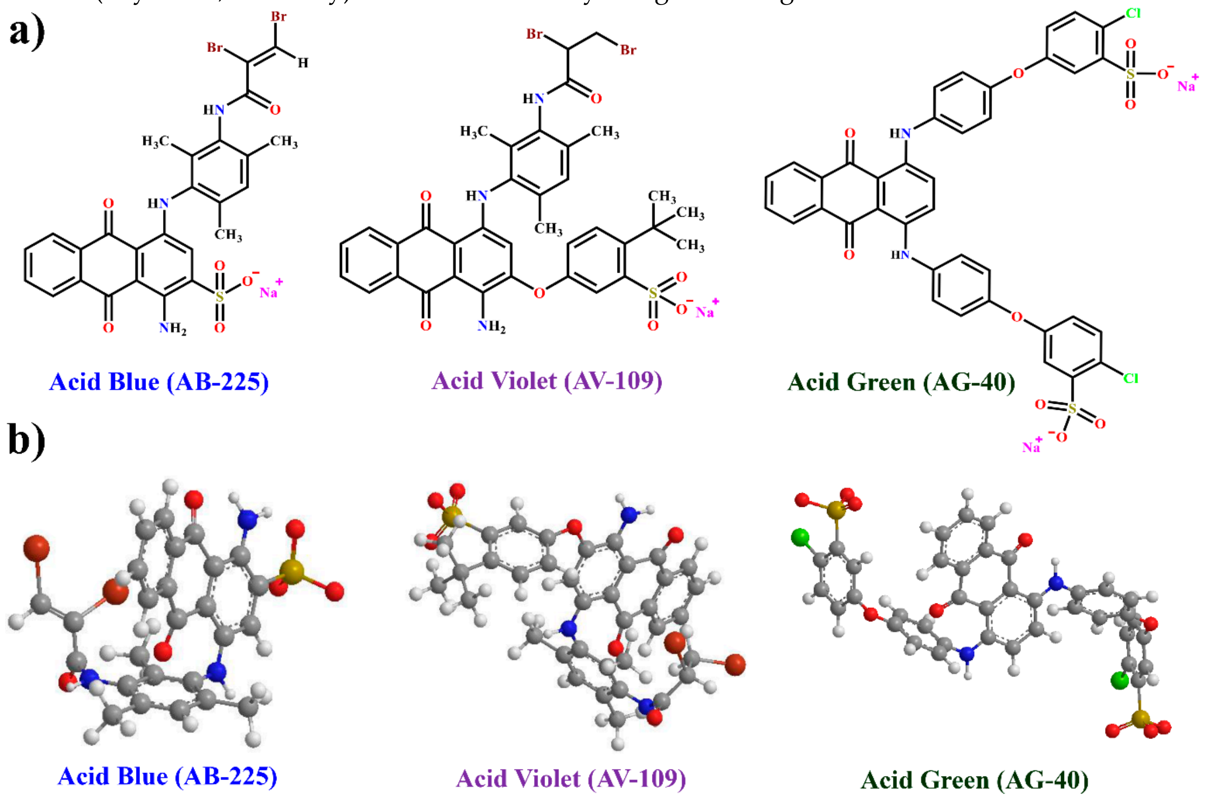

Figure S16. 2D a) and 3D structure b) of the dyes used in adsorption experiments.

S3.2. Syntheses of the MgAl-LDH and MgAl-LDH@SiO$_2$ adsorbent

S3.2.1. Synthesis of bio-silica particles

The literature [5] states that silica particles made from rice husks have been prepared. After washing the rice husk in water to get rid of any contaminants, it was dried. Rice husk (100 g) was treated for three hours at 80 °C in 400 mL of 10% H_2SO_4. After that, distilled water (DW) was used to wash the rice husk until the pH reached 7. After three hours of drying at 50 °C, it was heat-treated for four hours at 800 °C in an oven to produce silica white powder. The H_2SO_4 solution was essential in cleaning the rice husk of contaminants and halting the development of black particles [5].

S3.2.2. Synthesis of MgAl-LDH adsorbent

The coprecipitation approach was used to synthesize MgAl-LDH (molar ratio Mg: Al = 3: 1) from aqueous solutions under air conditions using $MgCl_2{\times}2H_2O$ (0.45 mol) and $Al_2(OH)_5Cl{\times}2.5\ H_2O$ (0.15 mol) as precursors. A 1M NaOH solution was used to promote deposition until the pH reached 10, mixed for 10 h and left overnight. Afterward, the obtained materials were washed with deionized water (DI) (ultrasound treatment) and precipitated using centrifuge at 5000 o/min (three-step processes until neutral pH of washing was obtained). The results of textural properties determination are given in Table S19.

Table S19. Textural properties of MgAl-LDH.

Sample	S_p (m^2/g)	V_{total} (cm^3/g)	V_{meso} (cm^3/g)	V_{micro} (cm^3/g)	D_{sr} (nm)	D_{max} (nm)
1:1	176.4	0.3498	0.3398	0.0502	6.0	3.6
2:1	176.6	0.3409	0.3252	0.0541	6.7	3.7
3:1	201.4	0.4171	0.4067	0.0631	7.0	4.8

S_p - specific surface area; V_{total} - total pore volume; V_{meso} – mezopore volume, pore ranged between 2.0 – 300 nm, V_{micro} – micropore volume, D_{sr} – average pore diameter; D_{max} – pore diameter occupying largest pore volume

The MgAl-LDH with a Mg:Al molar ratio of 3:1 showed beneficial textural properties and adsorption capacities, and, thus, it was selected for further experiments. Also, surface area of b-SiO$_2$ was 6.9837 m^{2}g^{-1}, V_{total} (cm^3/g) 0.0191 cm^{3}g^{-1}, V_{meso} (cm^3/g) 0.0226 cm^{3}g^{-1} and D_{sr} (nm) 9.958 nm.

S3.2.3. Optimization of MgAl-LDH@SiO$_2$ synthesis

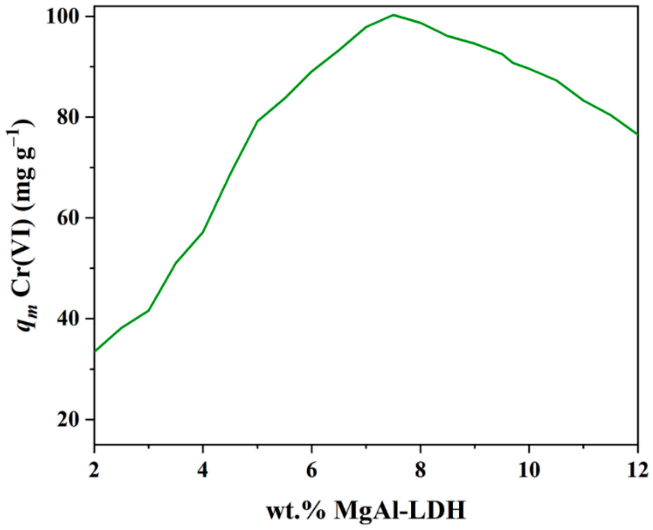

Figure S17. Results of the optimization of MgAl-LDH@SiO$_2$ synthesis: q_m (Cr(VI), mg g^{-1}), *versus* wt.% of MgAl-LDH deposit obtained at stepwise LDH precipitation.

S3.2.4. Modification of MgAl-LDH@SiO_2 with amino-silane (amino-MgAl-LDH@SiO_2)

MgAl-LDH modification with 3-Aminopropyltriethoxysilane was performed in a following way: 20 g of MgAl-LDH was placed in a two-necked flask (500 cm^3) with 3.2 mL of amino-silane, and 300 mL of toluene. The flask was placed on a magnetic stirrer and heated to 70 °C. The reaction lasted for 24 h at 70 °C and 300 μL of 96% ethanol was added to the flask a 2h in three portions. After the reaction, the solution is filtered through PVDF filter paper (45 nm pore size), washed with toluene, and dried for 4 h at 50 °C [16].

S3.3. Synthesis of bio-based unsaturated polyester resin (b-UPR)

S3.3.1. Synthesis of maleic anhydride (MA) from biobased resources

The synthesis of renewable maleic anhydride (MA) from 5-[(formyloxy)methyl] furfural (FMF) was developed using air as an oxidant with the help of $MnO_2/Cu(NO_3)_2$ as a catalyst. Synthesis conditions followed the work of [2,5,17].

S3.3.2. Synthesis of b-UPR

The precursor glycolysate, used for b-UPR synthesis, was obtained from ground post-consumer waste PET bottles. In a four necked flask (500 mL) equipped with reflux condenser, thermometer, nitrogen inlet tube and Dean-Stark separator was added PG (51 g, 0.67 mol) and 1 g Fascat 4100 catalyst following by gradual addition of PET (130 g, 0.67 mol) at 180 ºC for 2 h, and followed heating at 215-220 ºC for 6 h. During cooling to 115 °C the product was subjected to vacuum to remove excess of reactant and followed by bio-based MA (66 g, 0.67 mol) and hydroquinone (HQ) (0.02 g) addition and heating for 1 h, at same temperature. Increase of temperature to 150 ºC, at a rate of 10 ºC/min, was followed by TBT (0.3 wt.%) and xylene addition to enable azeotropic water removal. Heating was continued to reach 210-220 °C and held until almost stoichiometrically generated reaction water was separated (acid value of b-UPR < 20). After, the reaction mixture was cooled to 120 °C, 0.02 g of HQ in 0.2 mL of absolute ethanol was added, and a vacuum was applied to remove low boiling compounds. At 90 °C, the b-UPR obtained was dissolved in styrene (the final dilution was set at 40 wt.% of styrene), and used for composites production [5].
The physico-chemical characteristics of b-UPR is given in Table S20.

Table S20. Physico-chemical properties of b-UPR [5].

Parameter	Value	Unit	Method
Appearance	Transparent yellow	-	-
Non-volatile material content	60±2	wt.%	ISO 3251
Acid/Hydroxyl value (AV/HV)	12/25	mg KOH/g	ISO 2114/
Gel time, 20 ºC: 2 wt.% MEKP-50	25-30	Min	ISO 2535
Temperature of exotherm peak	110-120	ºC	ISO 2535
Density	1.37±0.09	kg m^{-3}	ISO 2811
Iodine value	51	-	Wijs Method

The viscosity of the b-UPR, 60 wt.% in styrene, was measured at 25 °C using a Ford cup 4 (ASTM D1200). Calculated molar mass, M_n was 3032 g mol^{-1}. The gel time of b-UPR, as determined by the cure exotherm, was 25-30 min, while viscosity of a 60 wt.% solution of b-UPR in styrene measured with the Ford viscosity cup ranged from 85 to 93 s.

S3.4. Adsorption/desorption study in a batch and fixed-bed column system

S3.4.1. Batch adsorption experiments

Isotherm, thermodynamic and kinetic parameters were determined from the results of comprehensively performed adsorption experiments. In brief, 10 mL of Cr(VI) (10 mg dm^{-3}) and dyes solutions (25 mg dm^{-3}) with a different mass of adsorbents of either MgAl-LDH or MgAl-LDH@SiO_2 (1, 2.5, 5, 7.5 and 10 mg), was equilibrated for 90 min at optimal pH 6. The process of adsorption lasted 90 min at different temperatures (25 °C, 35 °C, and 45 °C). The initial pH solution was adjusted by 0.1 M KOH and 0.1 M HCl solution. A mixer with digital heating, Drybath Shaker/Thermo SCIENTIFIC Laboratory Shaker, United States of America, was used in a batch system experimentation. After equilibration, the eluent was separated from the adsorbent by filtration using a 0.22 μm PTFE syringe filter. The collected filtrate was analysed using atomic absorption spectrometry (AAS), Perkin Elmer PinAAcle 900T, United States of America.

The process of establishing the adsorption equilibrium of dyes/adsorbent was monitored for 60 min at a wavelength of 630 nm (AB-225) and of 590 nm (AV-109) and 615 nm (AG-40), using UV-Vis Spectrophotometer 1800, Shimadzu, Japan; The time-dependent batch experiments performed at different time intervals (5, 10, 15, 20, 30, 45 and 60 min) and three different temperature was used for calculation kinetic and activation parameters.

The adsorption capacity at equilibrium, q_e (mg g^{-1}), was calculated based on the difference between the initial (C_i, mg L^{-1}) and final adsorbate concentration (C_e, mg L^{-1}) using Eq. (S13) [2]:

$$\mathrm{qe} = \left(\frac{(\mathrm{Ci-Ce})}{\mathrm{W}}\right) \times \mathrm{V} \tag{S13}$$

and the percentage of adsorbate removal was calculated using Eq. (S14) [2]:

$$\%Removal = \left(\frac{(Ci-Ce)}{Co}\right) \times 100 \tag{S14}$$

where C_i and C_e (mg L^{-1}) are the initial concentration and final concentration of the adsorbate, V is the volume of the solution (L) and W is the mass of the adsorbent (g).

S3.4.2. Bed column experiments

The experiments were performed in a glass column with a diameter of 0.7 cm and a height of 12 cm. Glass wool was placed at the bottom of the column, primarily to support a layer of adsorbent and to prevent the test material (0.78 g and 0.40 g of MgAl-LDH@SiO_2) from being displaced during the adsorption/desorption experiments at feed concentrations of 25 (high inlet) and 1 (low inlet) mg L^{-1}.

The concentration ratio at the breakthrough point at the outlet (Ce) was Ce/Ci>95%, as recommended in the literature [18]. On the introduced two-layer, another layer of glass wool was placed to prevent the formation of a funnel and channels in the layer during the introduction of the solution. The experiments were performed with solutions containing studied anions with variations of the inlet flow Q = 0.5, 1.0 and 1.5 cm^3 min^{-1}.

Among the packed-bed dynamics models are the Bohart-Adams (B-A) and Yoon-Nelson (Y-N) commonly used for large-scale operations. In liquid adsorption systems, these models predict the dynamic characteristics of the column and provide a deeper overall elucidation of this process.

The Bohart-Adams model [19] assumes that the adsorption rate is proportional to both the adsorbent's residual capacity and the adsorbing species' concentration. The linear form of this model is given by the following Eq. (S3). The Yoon-Nelson model [20], the same in this case, claims that the rate of reduction of the adsorption probability for each adsorbate molecule is proportional to the adsorbate adsorption probability and the probability of the adsorbate breakthrough point (Eq. S4). The linear forms of models are given by Eq. (S15, S16):

$$ln\left(\frac{C0}{C}-1\right) = KBA\left(N0\frac{Z}{U0}-C0t\right) \quad (S15)$$

$$ln\left(\frac{C0}{Ct}-1\right) = KYN(Q-t) \quad (S16)$$

Where C_o (mol dm^{-3}) is the initial concentration, C_t (mol dm^{-3}) is the concentration of effluent at time t, t (min) is flow time, Z (cm) is bed depth of column, N_0 (mg dm^{-3}) is maximum pollutant uptake capacity per unit volume of the adsorbent column, U_0 (cm min^{-1}) is the linear velocity of influent pollutant solution, K_{AB} (dm^3 mg^{-1} min^{-1}) is the kinetic constant, Θ (min) is the time required for 50% breakthrough, K_{YN} (min^{-1}) is the Yoon-Nelson rate constant, q_0 is the equilibrium adsorption per unit adsorbent mass (mg g^{-1}), m represents the mass of the adsorbent in column (g), Q represents the aqueous flow solution (wastewater) through the column (cm^3 min^{-1}).

S3.4.3. Desorption study

Several desorption agents were used: 2 wt.% NaOH/2 wt.% NaCl, 4 wt.% NaCl, 3 wt.% $CaCl_2$, and 4 wt.% Na_2SO_4.The most effective was 2 wt.% NaOH/2 wt.% NaCl and 1 wt.% NaOH/2 wt.% NaCl regenerator, while 4 wt.% NaCl, 3 wt.% $CaCl_2$, and 4 wt.% Na_2SO_4 showed somewhat lower efficiency.

In order to evaluate the desorption from MgAl-LDH and MgAl-LDH@SiO_2, five cycles of desorption were performed in a bed column according to two procedures:

a) High dye inlet concentration, C_i=25 mg dm^{-3}, in a adsorption step (experiments described in subsection S2.4.1) using 0.41 g of MgAl-LDH@SiO_2 in a desorption experiments by passing 200 mL of 2 wt.% NaOH/2 wt.% NaCl through the column (Table S13), at room temperature, Q = 0.5 cm^3 min^{-1} for dyes and Cr(VI) desorption and 1.0 cm^3 min^{-1} for AG-40.

b) Low dye inlet concentration, C_i=1 mg dm^{-3}, in a adsorption step (experiments performed at low inlet concentration, subsection S2.4.1), and 0.40 g of MgAl-LDH@SiO_2, desorption experiments was performed by passing 3 dm^3 for dyes and 1 dm^3 for Cr(VI) of 1 wt.% NaOH/2 wt.% NaCl through the column (Q = 0.5 cm^3 min^{-1}) (Table 6) at room temperature and desorption, respectively. Two concentrations were selected to study effectiveness of MgAl-LDH@SiO_2) absorption/desorption processes at high (effluent water from textile industry before discharging into watercourses) and low concentration (after discharging into river) trying to respond to needs for solving environmental challenge related to pollution which is generated from Serbian textile industry.

After each desorption cycle, the solutions were filtered and the remaining material was used for the next adsorption/desorption cycle. The effluent was separated from the adsorbent by vacuum filtration through a PTFE filter with a pore diameter of 0.22 μm. All collected effluent water was analysed using atomic absorption spectrometry (AAS). The results were expressed as the adsorption capacity, the amount of desorbed material, the efficiency of regeneration of the adsorbent, the concentration of the pollutant in the effluent, and the residue (irreversibly bound pollutant) after each adsorption-desorption cycle. The concentration of dyes in the liquid phase at the different desorption times was measured by UV-Vis's absorbance. The adsorbent regeneration efficiency was calculated with the following Eq. (S17) [2] :

$$(\%RE) = \frac{q_r}{q0} \times 100 \quad (S17)$$

The terms q_0 and q_r refer to the adsorption capacities of the adsorbents (measured in mmol g^{-1} or mg g^{-1}) before and after the desorption process, respectively. Desorption efficiency indicates the percentage of the desorbed pollutant relative to the amount of adsorbed pollutant per unit mass of the adsorbent [21].

Concentration of the effluent solution was adjusted at working one applicable in a subsequent experiment of either enzymatic decolorization or photocatalytic degradation.

S3.5. Technologies developed for desorbed pollutant and exhausted adsorbent disposal

S3.5.1. Disposal of exhausted adsorbent

Exhausted adsorbent (50 g), after five adsorption/desorption cycles, was submerged into 100 cm^3 0.1 mol dm^{-3} H_2SO_4 until MgAl-LDH deposit was dissolved (concentration determined using AAS after three sequential washing to obtain <20 ppb of both ions). Silica nanoparticle washing with DW and drying provides similar mass of silica carrier (synthesis 90.2%, after LDH dissolution 89.9%).

Silica nanoparticles was used either as reinforcement in b-UPR or modified with vinyl silane to obtain reactive reinforcement in b-UPR based composites [5]. The results of the mechanical testing of corresponding composites are given in Table S15.

Into washing solution of 100 cm^3 (containing 0.61 mol dm^{-3} $MgSO_4$ and 0.20 mol dm^{-3} $Al_2(SO_4)_3$) was slowly added (drop-wisely) 1 mol dm^{-3} NaOH solution until attainment of pH 6.5, and white cloudy solution was left overnight. After centrifugation and three washing with DI water the filtration cake was transferred to autoclave with addition 100 mL of DI water and heated at 180 ºC for 4 h. The precipitate was filtrated and dried at 105 ºC for 3 h.
Residual water washing was additionally adjusted with 1 mol dm^{-3} NaOH to pH ~10.5, left overnight and after repeated centrifugation and three washing with DI water was dried at 105 ºC for 3 h.

S3.5.2. Preparation of immobilized enzyme on amino-MgAl-LDH@SiO_2 support

- *Immobilization procedure*

Immobilization of HRP was carried out by adding 20 mg of amino-MgAl-LDH@SiO_2 into 1 mL of HRP solution (different concentration of HPR were used) in phosphate buffer (100 mM, pH 7.0), and the suspension was put on roller shaker for 24 h at room temperature. Immobilized enzyme was separated from the supernatant by centrifugation (5 min 12 000 rpm) and washed 2 times with immobilization buffer.

- *Protein assay using the Bradford method*

Protein content was determined using the method of Bredford [22]. The difference between the initial protein concentration in the supernatant at the start of the immobilization process and in the supernatant after immobilization process is finished represents the mass of bound protein (protein loading mg/g of support). The protein immobilization yield was calculated after protein content determination in the initial solution and separated supernatants [23].

- *Activity assay (determination of activity in reaction with commercial substrate pyrogallol)*

Activity of the free and immobilized HRP was measured using standard substrates, hydrogen peroxide and pyrogallol. The reaction mixture for measuring the activity of free enzyme consisted of 1 mL of 13 mM pyrogallol in the Na-phosphate buffer pH 7.0, 10 μL of 3% hydrogen peroxide, and 10 μL of diluted enzyme solution [24]. The reaction rate was monitored spectrophotometrically at 420 nm by following the change in absorbance values every 30 s for 3 min [25]. The reaction mixture for measuring the activity of immobilized HRP consisted of 10 mL of 13 mM pyrogallol prepared in the potassium phosphate buffer pH 7.0, where 20 mg of immobilized enzyme was suspended. After adding 30 μL of hydrogen peroxide, magnetic stirrer was set to the maximum and the reaction rate was recorded every 60 s for 3 min. One unit of the activity was defined as the amount of peroxidase that will form 1.0 mg of purpurogallin from pyrogallol in 20 s at pH 7.0 and 20 °C.

$$\text{Activity of immobilized enzyme}\left(\frac{\text{IU}}{\text{gsupport}}\right) = \text{R} \times \text{A} \times \frac{12}{\text{me}} \times 100 \quad \text{(S18)}$$

When m_e is the mass of immobilized enzyme (g), A is the absorbance at 420 nm

- *Support activation procedure*

20 mg of amino-MgAl-LDH@SiO_2 was suspended in 0.5 mL 1% glutaraldehyde solution (GA) and gently mixed on roller shaker at room temperature for 2 h [26]. After the activation reaction was finished excess of glutaraldehyde was washed several times with distilled water. Prepared glutaraldehyde activated support was used for the HRP immobilization. The same immobilization procedure was applied as in the case of amino-MgAl-LDH@SiO_2. After immobilization was finished the activity of immobilized enzyme was determined.

- *Desorption assay*

To examine the nature of contacts and the extent of desorption between the treated support and horseradish peroxidase, the following protocol was employed for treating immobilized preparations with 1 M $CaCl_2$: 20 mg of HRP immobilized on Ga-amino-MgAl-LDH@SiO_2 was subjected to $CaCl_2$ treatment for 1 hour at a temperature of 25 °C and a speed of 150 rpm in an orbital shaker. Subsequently, the immobilized enzyme was washed with distilled water three times, and its activity in reaction with pyrogallol and H_2O_2 was then measured.

- *Reusability study*

To test the recycling capacity of immobilized peroxidase (HPR), the immobilized preparation was employed in sequential dye decolorization reactions (each lasting 4 h). Following each cycle, the dye solution was withdrawn and absorbance was measured, and the immobilized enzyme was rinsed twice with buffer. An identical amount of dye stock solution (25 mL, concentration of 25 mg L^{-1}) was added to each tube to start the next cycle.

S3.5.3. Dye decolorization procedure

Textile dye decolorization was evaluated by incubating the adsorbed and covalently immobilized HRP with equal activity in AV-109 solution (C_i=25 mg L^{-1}) at pH 4.0 under 25 °C. Influence of hydrogen peroxide concentration on the decolorization was examined by varying the hydrogen peroxide concentration in the range 0.05-0.5%. In defined time intervals aliquots were collected from the reaction mixtures and the residual dye amount was followed using a UV–Vis's spectrophotometer (UV Shimadzu 1700, Shimadzu Corporation, Kyoto, Japan) at maximum wavelength for the tested dye ($\lambda_{AV\text{-}109}$ = 590 nm, $\lambda_{AB\text{-}225}$ = 630 nm).
Decolorization percentage was calculated using the following mathematical expression [25]:

$$\text{Decolorization degree (\%)} = \frac{A0 - At}{A0} * 100 \qquad \text{(S19)}$$

Where Ao is the initial absorbance of the dye and the absorbance of the dye with time (At).

S3.5.4. Photocatalytic experiment

Photodegradation was initially tested with higher dye concentrations in the effluent, after the first desorption cycle (Table S11). Such procedure required long time of exposure not applicable in a real condition (over 5 days of exposure). Moreover, low desorption efficiency indicates low applicability of such methodology. Optimization of the photodegradation time required short time which mean that lower C_{eff} should be obtained. The degradation kinetics of the dye molecule were investigated using UV/Vis's spectroscopy to track the shift in the intensity of the absorption peaks measured at 615 nm (AG-40), for 210 min using ZnO catalyst with a concentration of 0.08 g L^{-1}). Initial value of mixed effluent solution and diluted to 22.7 mg L^{-1} of AG- 40 was 576 mg O_2 L^{-1}.

The photodegradation experiments were conducted in a Pyrex glass photoreactor equipped with a water-cooling jacket, a magnetic stirrer, and an Osram Ultra Vitalux lamp with a nominal wattage of 300 W (UVA 315-400 nm: UVB 280-515 nm = 13.6 W: 3 W, the manufacturer's data). The apparatus was protected from sunlight by placing in a dark box.

The prepared catalyst and dye solutions were allowed to stir in the dark for 30 min (150 rpm), and after the dispersion was exposed to UV-Vis (300-800 nm) irradiation at 20 cm distance between reactor and lamp. The reactor is constantly cooled to keep constant temperature at 20 ºC.

The aliquot (3 mL) at defined time intervals (Figure 7) were collected, subjected to centrifugation (6000 rpm) and filtered through PVDF 0.22 μm pore size syringe filters.

As the maximum permissible concentrations (MPC) are not defined for studied dyes in the national regulatory documents, the quality of the treated water was evaluated by determination of chemical oxygen demand (COD). Experiments were conducted to attain regulatory requirement for COD Limit values of wastewater emissions from plants and plants for textile processing and production set at 200 mg O_2 L^{-1} (Decree on limit values of emissions of polluting substances in introductions and deadlines for their achievement, Official Gazette of the Republic of Serbia No. 67/2011 and 48/2012). Initial value of mixed effluent solution and diluted to 22.7 mg L^{-1} of AG- 40 was 576 mg O_2 L^{-1}.

The efficiency of the dye photodegradation was calculated according to Eq. (S20) [27]:

$$\eta = \frac{(Co-Ct)}{Co} \times 100 = \frac{(Ao-At)}{Ao} \times 100 \quad \text{(S20)}$$

where η is the degradation rate (%), C_0 represents the initial concentration of dye, Ct stands for dye concentration after the reaction, A_0 indicates initial absorbance, and A_t shows the absorbance of the system after the appropriate degradation time [28].

The Eq. (S21) was used for the determination of the pseudo-first order rate kinetic parameters [29–31]:

$$ln\left(\frac{Ct}{C0}\right) = -K * t \quad \text{(21)}$$

where C_0 and C are concentrations of monitored analyte at the start and in the certain moment of observation, respectively, t is the period and K is the apparent rate constant. The half-time of the pseudo-first order rate equation was calculated using Eq. (S22) [31]:

$$\mathrm{t}_{1/2} = \frac{\ln 2}{\mathrm{K}} \quad \text{(S22)}$$

To rate constant k' was calculated according to Eq (S23) [32]:

$$\mathrm{k}' = \frac{\mathrm{K}}{\mathrm{m}} \quad \text{(S23)}$$

where k' is equal to the ratio of K (apparent rate constant; s^{-1} or min^{-1}) and m (the exact catalyst dosage in g or mg used).

S3.5.5. Disposal of Cr(VI)

Among many Cr(VI) precipitation/solidification methods as: chrome yellow by precipitation of chromate ion using lead (in acidic medium (pH<4) [33] provide efficient way of Cr(VI) removal in effluent water. In the past it was used as pigment in paint production and corrosion protection, but nowadays this alternative is of lower value because it has limited application. Chromate conversion coating was widely used for corrosion protection of reactive metal such as zinc and aluminium for aeronautical application [34]. Nowadays, it is banned in EU, and allowed to be used for specific application such as aeronautical application. Countries that are not in the European Union still use chromate conversion coatings. The most valuable method was found to be reduction to environmentally-friendly Cr(III) form, and subsequent precipitation and dehydration to Cr(III)-oxide provided non-harmless product. An efficient method was based on zero valent iron use as reducing agent [35] at pH 2, C_{eff} [Cr(VI)] = 26.2 mg L^{-1}, 15 g L^{-1} ZVI (finely ground), t = 15 min provide 100% conversion Cr(VI) to Cr(III). Further, adjusting of solution pH to 9 provide precipitation of the chromium and iron (III)-hydroxides. Residual Cr(VI) of 2.5 μg L^{-1} (limit values 0.1 mg L^{-1}) in solution and TCLP (Toxicity characteristic leaching procedure) showed evaluability of the applied technology.

S3.6. Characterization methods

- *Melting points* were determined on a SMP30 Melting point apparatus.

- *Elemental analyses* (C, H, N, S) of percussor were performed by the standard micro methods using the ELEMENTAR Vario EL III CHNS/O analyser.
- *Nuclear magnetic resonance spectroscopy (^1H and ^{13}C NMR) spectra* were recorded at room temperature in DMSO-d_6 on a Bruker Avance III 500 spectrometer equipped with a broad-band direct probe. Chemical shifts are given on δ scale relative to tetramethylsilane (TMS) as an internal standard in ^{1}H NMR spectra, while to residual solvent signal in ^{13}C NMR.
- *CEC determination*

In order to determine cation exchange capacity (CEC), mass concentrations of sodium, potassium, magnesium, calcium, and iron were done for all samples [36].

- *Surface Morphology analysis*

Morphologies of MgAl-LDH@SiO_2, particles before and after adsorption, were examined using a 20 kV MIRA3 TESCAN Field-Emission Scanning Electron Microscopy (FE-SEM/EDS).

- *Transmission electron microscope*

Transmission electron microscope (TEM), model JEM-1400, with an accelerating voltage of 120 kV was used for investigation microstructural analysis of MgAl-LDH@SiO_2 particles.

- *X-ray diffraction analysis (XRD)*

Crystal phases of MgAl-LDH@SiO_2 in a Bragg–Brentano geometry was performed using CuK radiation (λ= 1.5418) and step-scan mode (range: 10-80 °C, step-time: 0.50 s, step-width: 0.02°) on a Rigaku X-ray diffractometer.

- *Fourier Transform Infrared Spectroscopy (FTIR)*

FTIR spectra were collected with a Nicolet™ iS™ 10 FT-IR Spectrometer (Thermo Fisher SCIENTIFIC) with Smart iTR™ Attenuated Total Reflectance (ATR) Sampling accessories. The spectra were recorded in the range 4000-400 cm^{-1}, in 20 scans mode, and at a resolution of 4 cm $^{-1}$

- *X-ray photoelectron spectroscopic analysis (XPS)*

XPS analysis of the samples was carried out on SPECS Systems with XP50M X-ray source for Focus 500 and PHOIBOS 100 energy analyser using a monochromatic Al Kα X-ray source (1486.74 eV) at 12.5 kV and 16 mA. The sample was fixed onto an adhesive copper foil to provide strong mechanical attachment and good electrical contact. Survey XPS spectrum (0–1000 eV BE) was recorded with a constant pass energy of 40 eV, energy step of 0.5 eV, and the dwell time of 0.2 s, while high resolution XPS spectra of the corresponding lines were taken with a pass energy of 20 eV, energy step of 0.1 eV and a dwell time of 2 s. The XPS spectra were collected by SpecsLab data analysis software, and analysed using CasaXPS software package. A standard Shirley background is used for all sample spectra.

- *Determination of Chemical oxygen demand (COD)*

Additionally, in order to define criteria for the successfulness of the adsorption process, in default of regulation, international and national data, the most relevant approach was to use limit values given by the Serbian national document related to limit values for the emission of pollutants at the site of the discharge into surface water: *Decree on limit values for the emission of pollutants into water and deadlines for their achievement* (RS Gazette, no. 67/2011, 48/2012 and 1/2016). Maximum allowable value for discharge water: COD = 200 mg O_2 dm^{-3}; BOD = 30 mg dm^{-3} and TOC= 60 mg dm^{-3}.
The standard method ISO 6060 was used for the determination of the chemical oxygen demand (COD). Water samples after treatment were subjected to spectrophotometric tests, with the aim of determining COD values. Mercury (II) sulphate was used as a masking agent for chloride. The devices used for COD determination:

1. Lovibond MD 600 spectrophotometer, Tintometer GmbH,
2. Lovibond RD 125 sample heating apparatus, Tintometar GmbH

All the solution with concentration exceeding the limit of detection was diluted prior to analysis.

- *Quantum yield*

As a measure of the efficiency of all studied processes, the measurement of quantum yield was approached. For that purpose, $K_3[Fe(C_2O_4)_3]$ as a chemical actinometer was prepared [37]. A

synthesized actinometer was used for measuring photons absorbed per unit of time into a particular volume.

- *pH_{PZC} determination*

Point of zero charge - pH_{PZC} represents the pH value at which the sum of the negative and positive charges is equal or, in the other word, the net charge around the particle surface is equal to zero. The pHpzc value depends on the chemical reagents used for synthesis and production method, ionic strength of the solution, type, and number of surface functionalities. Thus, pH-dependent behavior of MgAl-LDH and MgAl-LDH@SiO_2 was of significant importance to be studied in order to understand system response at different pH.

The pHpzc value was determined using a pH meter HI-2210-02 Bench Top, HANNA instruments, Hungary, with an instrument accuracy of ± 0.01. A set of pH solutions in the range of 2 - 12 was made, using KNO_3 at concentrations of 0.1, 0.01, and 0.001 M, respectively. The 50 mL of KNO_3 solution was allowed to equilibrate for 24 h with 100 mg of adsorbent. Each sample is adjusted to a specific initial pH value, and after 24 h the pH measurement procedure is repeated and recorded as the final pH value.

- *Image analysis*

Software Image-Pro Plus 6.0 (Media Cybernetics, USA) was used to obtain the statistical data of the distribution of diameters of the MgAl-LDH@SiO_2.

- *Amino group determination*

Quantification of the amino group, present on amino-MgAl-LDH@SiO_2 was determined *via* "back" (indirect) titration as follow: 100 mg of amino-MgAl-LDH@SiO_2 adsorbent was placed in 100 cm^3 0.01 mol dm^{-3} HCl and treated ultrasonically for 15 min. Materials were filtered, and 10 cm^3 of supernatant was titrated with a standard solution of 0.01 mol dm^{-3} KOH in the presence of methyl orange indicator. The acid value (*AV*) was evaluated according to ASTM D3644.

- *Leaching test*

Leaching test was performed to obtain an information on the release of hazardous chemical constituents/pollutants from stabilized materials which include pollutants from effluent water. The experimental procedure was performed according to the Toxicity Characteristic Leaching Procedure (TCLP) and the EN12457-2003 [2]. Standard using acetic acid and deionized water as an extraction fluid.

- *Flammability testing*

The UL-94 vertical test was carried out using specimen made of b-UPR/Mg or Al-hydroxide composites. The samples were created in moulds measuring 120 mm in length, 10 mm in width, and 4 mm in thickness. The test describes the material's tendency to extinguish or spread the flame after the ignition of the composite. After applying a blue flame of 20 mm height to the bottom edge of the vertical sample for 10 s, the post-flame time required to extinguish the burning material is recorded (Figure S18). The flame is applied for another 10 s, and the time it takes to extinguish is recorded. There is a chance that the sample will leak during the combustion test, igniting a piece of cotton placed beneath the standard apparatus.

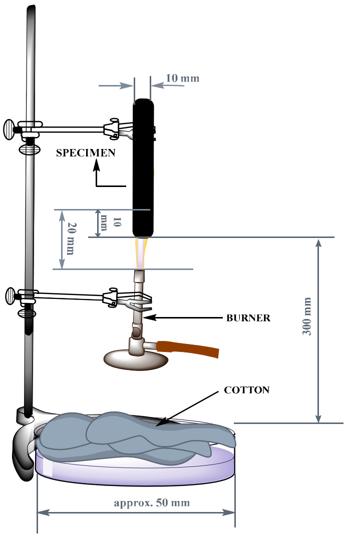

Figure S18. Schematic illustration of UL-94V flammability test.

A Bunsen burner was used to light the tubes, which were suspended vertically over surgical cotton. Flammability levels range from V-0 to V-2 (Table S2). The sample's lower end was exposed to the flame for 10 s before being removed. It was a time stamp. If the flame time is less than 10 s, the sample is classified as V-0. This method designates combustible materials that cannot be classified as NC (no classification, complete combustion pattern).

Table 21. Classification of materials according to the UL-94V test [2].

**Criteria conditions**	**V-0**	**V-1**	**V-2**
After flame time for each individual flaming	≤ 10 s	≤ 30 s	≤ 30 s
After flame and glow time for each individual specimen, after the second flaming	≤ 30 s	≤ 60 s	≤ 60 s
Total after flame time for any condition set	≤ 50 s	≤ 250 s	≤ 250 s
Cotton indicator ignited by flaming drops	No	No	Yes
After flame and afterglow time of any specimen up to the holding clamp	No	No	No

The testing was performed using three specimens.

References

1. Lombardo, S.; Thielemans, W. Thermodynamics of Adsorption on Nanocellulose Surfaces. *Cellulose* **2019**, *26*, 249–279, doi:10.1007/s10570-018-02239-2.
2. Knežević, N.; Milanović, J.; Veličković, Z.; Milošević, M.; Vuksanović, M.M.; Onjia, A.; Marinković, A. A Closed Cycle of Sustainable Development: Effective Removal and Desorption of Lead and Dyes Using an Oxidized Cellulose Membrane. *J. Ind. Eng. Chem.* **2023**, doi:10.1016/j.jiec.2023.06.041.
3. Jamal, F.; Singh, S.; Qidwai, T.; Singh, D.; Pandey, P.K.; Pandey, G.C.; Khan, M.Y. Catalytic

Activity of Soluble versus Immobilized Cauliflower (Brassica Oleracea) Bud Peroxidase-Concanavalin A Complex and Its Application in Dye Color Removal. *Biocatal. Agric. Biotechnol.* **2013**, *2*, 311–321, doi:10.1016/j.bcab.2013.05.005.

4. Jovanović, A.; Stevanović, M.; Barudžija, T.; Cvijetić, I.; Lazarević, S.; Tomašević, A.; Marinković, A. Advanced Technology for Photocatalytic Degradation of Thiophanate-Methyl: Degradation Pathways, DFT Calculations and Embryotoxic Potential. *Process Saf. Environ. Prot.* **2023**, *178*, 423–443, doi:10.1016/j.psep.2023.08.054.
5. Embirsh, H.S.A.; Stajčić, I.; Gržetić, J.; Mladenović, I.O.; Anđelković, B.; Marinković, A.; Vuksanović, M.M. Synthesis, Characterization and Application of Biobased Unsaturated Polyester Resin Reinforced with Unmodified/Modified Biosilica Nanoparticles. *Polymers (Basel).* **2023**, *15*, 3756, doi:10.3390/polym15183756.
6. Pan, X.; Zhang, M.; Liu, H.; Ouyang, S.; Ding, N.; Zhang, P. Adsorption Behavior and Mechanism of Acid Orange 7 and Methylene Blue on Self-Assembled Three-Dimensional MgAl Layered Double Hydroxide: Experimental and DFT Investigation. *Appl. Surf. Sci.* **2020**, *522*, 146370, doi:10.1016/j.apsusc.2020.146370.
7. Extremera, R.; Pavlovic, I.; Pérez, M.R.; Barriga, C. Removal of Acid Orange 10 by Calcined Mg/Al Layered Double Hydroxides from Water and Recovery of the Adsorbed Dye. *Chem. Eng. J.* **2012**, *213*, 392–400, doi:10.1016/j.cej.2012.10.042.
8. Abbasi, M.; Sabzehmeidani, M.M.; Ghaedi, M.; Jannesar, R.; Shokrollahi, A. Synthesis of Grass-like Structured Mn-Fe Layered Double Hydroxides/PES Composite Adsorptive Membrane for Removal of Malachite Green. *Appl. Clay Sci.* **2021**, *203*, 105946, doi:10.1016/j.clay.2020.105946.
9. Lu, L.; Li, J.; Ng, D.H.L.; Yang, P.; Song, P.; Zuo, M. Synthesis of Novel Hierarchically Porous Fe3O4@MgAl–LDH Magnetic Microspheres and Its Superb Adsorption Properties of Dye from Water. *J. Ind. Eng. Chem.* **2017**, *46*, 315–323, doi:10.1016/j.jiec.2016.10.045.
10. Zheng, X.; Liu, D.; Wen, J.; Lv, S. Nonthermal Plasma-Vulcanized Flower-like ZnS/Zn-Al Composites from Zn-Al Layered Double Hydroxides for the Adsorption-Photo-Reduction of Cr(VI). *Sep. Purif. Technol.* **2021**, *275*, 117934, doi:10.1016/j.seppur.2020.117934.
11. Li, X.; Shi, Z.; Zhang, J.; Gan, T.; Xiao, Z. Aqueous Cr (VI) Removal Performance of an Invasive Plant-Derived Biochar Modified by Mg/Al-Layered Double Hydroxides. *Colloid Interface Sci. Commun.* **2023**, *53*, 100700, doi:10.1016/j.colcom.2023.100700.
12. Zhao, J.; Huang, Q.; Liu, M.; Dai, Y.; Chen, J.; Huang, H.; Wen, Y.; Zhu, X.; Zhang, X.; Wei, Y. Synthesis of Functionalized MgAl-Layered Double Hydroxides via Modified Mussel Inspired Chemistry and Their Application in Organic Dye Adsorption. *J. Colloid Interface Sci.* **2017**, *505*, 168–177, doi:10.1016/j.jcis.2017.05.087.

13. Ren, S.; Wang, Y.; Han, Z.; Zhang, Q.; Cui, C. Synthesis of Polydopamine Modified MgAl-LDH for High Efficient Cr(VI) Removal from Wastewater. *Environ. Res.* **2022**, *215*, 114191, doi:10.1016/j.envres.2022.114191.
14. Dai, X.; Jing, C.; Li, K.; Zhang, X.; Song, D.; Feng, L.; Liu, X.; Ding, H.; Ran, H.; Zhu, K.; et al. Enhanced Bifunctional Adsorption of Anionic and Cationic Pollutants by MgAl LDH Nanosheets Modified Montmorillonite via Acid-Salt Activation. *Appl. Clay Sci.* **2023**, *233*, 106815, doi:10.1016/j.clay.2023.106815.
15. Zeng, B.; Wang, Q.; Mo, L.; Jin, F.; Zhu, J.; Tang, M. Synthesis of Mg-Al LDH and Its Calcined Form with Natural Materials for Efficient Cr(VI) Removal. *J. Environ. Chem. Eng.* **2022**, *10*, 108605, doi:10.1016/j.jece.2022.108605.
16. Perendija, J.; Veličković, Z.S.; Cvijetić, I.; Rusmirović, J.D.; Ugrinović, V.; Marinković, A.D.; Onjia, A. Batch and Column Adsorption of Cations, Oxyanions and Dyes on a Magnetite Modified Cellulose-Based Membrane. *Cellulose* **2020**, *27*, 8215–8235, doi:10.1007/s10570-020-03352-x.
17. Jia, W.; Si, Z.; Feng, Y.; Zhang, X.; Zhao, X.; Sun, Y.; Tang, X.; Zeng, X.; Lin, L. Oxidation of 5-[(Formyloxy)Methyl]Furfural to Maleic Anhydride with Atmospheric Oxygen Using α-MnO 2 /Cu(NO 3) 2 as Catalysts. *ACS Sustain. Chem. Eng.* **2020**, *8*, 7901–7908, doi:10.1021/acssuschemeng.0c01144.
18. Sotelo, J.L.; Rodríguez, A.; Álvarez, S.; García, J. Removal of Caffeine and Diclofenac on Activated Carbon in Fixed Bed Column. *Chem. Eng. Res. Des.* **2012**, *90*, 967–974, doi:10.1016/j.cherd.2011.10.012.
19. Bohart, G.S.; Adams, E.Q. SOME ASPECTS OF THE BEHAVIOR OF CHARCOAL WITH RESPECT TO CHLORINE. 1. *J. Am. Chem. Soc.* **1920**, *42*, 523–544, doi:10.1021/ja01448a018.
20. Yoon, Y.H.; Nelson, J.H. Application of Gas Adsorption Kinetics I. A Theoretical Model for Respirator Cartridge Service Life. *Am. Ind. Hyg. Assoc. J.* **1984**, *45*, 509–516, doi:10.1080/15298668491400197.
21. Perendija, J.; Veličković, Z.S.; Cvijetić, I.; Lević, S.; Marinković, A.D.; Milošević, M.; Onjia, A. Bio-Membrane Based on Modified Cellulose, Lignin, and Tannic Acid for Cation and Oxyanion Removal: Experimental and Theoretical Study. *Process Saf. Environ. Prot.* **2021**, *147*, 609–625, doi:10.1016/j.psep.2020.12.027.
22. Kielkopf, C.L.; Bauer, W.; Urbatsch, I.L. Bradford Assay for Determining Protein Concentration. *Cold Spring Harb. Protoc.* **2020**, *2020*, pdb.prot102269, doi:10.1101/pdb.prot102269.
23. Bebić, J.; Banjanac, K.; Rusmirović, J.; Ćorović, M.; Milivojević, A.; Simović, M.; Marinković, A.; Bezbradica, D. Amino-Modified Kraft Lignin Microspheres as a Support for Enzyme

Immobilization. *RSC Adv.* **2020**, *10*, 21495–21508, doi:10.1039/D0RA03439H.

24. Svetozarević, M.; Šekuljica, N.; Onjia, A.; Barać, N.; Mihajlović, M.; Knežević-Jugović, Z.; Mijin, D. Biodegradation of Synthetic Dyes by Free and Cross-Linked Peroxidase in Microfluidic Reactor. *Environ. Technol. Innov.* **2022**, *26*, 102373, doi:10.1016/j.eti.2022.102373.
25. Sekuljica, N.; Prlainovic, N.; Jovanovic, J.; Stefanovic, A.; Grbavcic, S.; Mijin, D.; Knezevic-Jugovic, Z. Immobilization of Horseradish Peroxidase onto Kaolin by Glutaraldehyde Method and Its Application in Decolorization of Anthraquinone Dye. *Hem. Ind.* **2016**, *70*, 217–224, doi:10.2298/HEMIND150220028S.
26. Chen, H.; Zhang, Q.; Dang, Y.; Shu, G. The Effect of Glutaraldehyde Cross-Linking on the Enzyme Activity of Immobilized &beta-Galactosidase on Chitosan Bead. *Adv. J. Food Sci. Technol.* **2013**, *5*, 932–935, doi:10.19026/ajfst.5.3185.
27. Yang, C.; Li, Q.; Tang, L.; Xin, K.; Bai, A.; Yu, Y. Synthesis, Photocatalytic Activity, and Photogenerated Hydroxyl Radicals of Monodisperse Colloidal ZnO Nanospheres. *Appl. Surf. Sci.* **2015**, *357*, 1928–1938, doi:10.1016/j.apsusc.2015.09.140.
28. Saeed, K.; Khan, I.; Park, S.-Y. TiO 2 /Amidoxime-Modified Polyacrylonitrile Nanofibers and Its Application for the Photodegradation of Methyl Blue in Aqueous Medium. *Desalin. Water Treat.* **2015**, *54*, 3146–3151, doi:10.1080/19443994.2014.912157.
29. Naghash-Hamed, S.; Arsalani, N.; Mousavi, S.B. The Catalytic Reduction of Nitroanilines Using Synthesized CuFe 2 O 4 Nanoparticles in an Aqueous Medium. *ChemistryOpen* **2022**, *11*, doi:10.1002/open.202200156.
30. Konstantinou, I.K.; Albanis, T.A. TiO2-Assisted Photocatalytic Degradation of Azo Dyes in Aqueous Solution: Kinetic and Mechanistic Investigations. *Appl. Catal. B Environ.* **2004**, *49*, 1–14, doi:10.1016/j.apcatb.2003.11.010.
31. Li, Y.; Wu, M.; Yang, D.; Zeng, H.; Zhang, T.; Shen, J.; Zhang, B.; Li, Q. Novel High Efficiency Layered Oxide Photocatalyst Li2SnO3 for Rhodamine B and Tetracycline Degradation. *Catalysts* **2019**, *9*, 712, doi:10.3390/catal9090712.
32. Naghash-Hamed, S.; Arsalani, N.; Mousavi, S.B. Facile Copper Ferrite/Carbon Quantum Dot Magnetic Nanocomposite as an Effective Nanocatalyst for Reduction of Para-Nitroaniline and Ortho-Nitroaniline. *Nano Futur.* **2022**, *6*, 045003, doi:10.1088/2399-1984/ac9a19.
33. Li, W.; Zhang, C.; Wei, X.; Zhang, H.; Han, M.; Sun, W.; Li, W. Efficient Resource Treatment of Hexavalent Chromium Wastewater Based on Lead Carbonate (Cerussite)-Induced Precipitation Separation. *Process Saf. Environ. Prot.* **2022**, *165*, 475–486, doi:10.1016/j.psep.2022.07.039.
34. Sahoo, P.; Das, S.K.; Paulo Davim, J. 3.3 Surface Finish Coatings. In *Comprehensive Materials Finishing*; Elsevier, 2017; pp. 38–55.

35. Prasad, P.V.V. V.; Das, C.; Golder, A.K. Reduction of Cr(VI) to Cr(III) and Removal of Total Chromium from Wastewater Using Scrap Iron in the Form of Zerovalent Iron(ZVI): Batch and Column Studies. *Can. J. Chem. Eng.* **2011**, *89*, 1575–1582, doi:10.1002/cjce.20590.
36. Daković, A.; Tomašević-Čanović, M.; Dondur, V.; Rottinghaus, G.E.; Medaković, V.; Zarić, S. Adsorption of Mycotoxins by Organozeolites. *Colloids Surfaces B Biointerfaces* **2005**, *46*, 20–25, doi:10.1016/j.colsurfb.2005.08.013.
37. Tomašević, A.; Mijin, D.; Radišić, M.; Prlainović, N.; Cvijetić, I.; Kovačević, D. V.; Marinković, A. Photolysis of Insecticide Methomyl in Various Solvents: An Experimental and Theoretical Study. *J. Photochem. Photobiol. A Chem.* **2020**, *391*, 112366, doi:10.1016/j.jphotochem.2020.112366.
